# Computational evaluation of exome sequence data using human and model organism phenotypes improves diagnostic efficiency

**DOI:** 10.1038/gim.2015.137

**Published:** 2015-11-12

**Authors:** William P. Bone, Nicole L. Washington, Orion J. Buske, David R. Adams, Joie Davis, David Draper, Elise D. Flynn, Marta Girdea, Rena Godfrey, Gretchen Golas, Catherine Groden, Julius Jacobsen, Sebastian Köhler, Elizabeth M. J. Lee, Amanda E. Links, Thomas C. Markello, Christopher J. Mungall, Michele Nehrebecky, Peter N. Robinson, Murat Sincan, Ariane G. Soldatos, Cynthia J. Tifft, Camilo Toro, Heather Trang, Elise Valkanas, Nicole Vasilevsky, Colleen Wahl, Lynne A. Wolfe, Cornelius F. Boerkoel, Michael Brudno, Melissa A. Haendel, William A. Gahl, Damian Smedley

**Affiliations:** 1 Undiagnosed Diseases Program, Common Fund, Office of the Director, National Institutes of Health, Bethesda, Maryland, USA; 2 Genomics Division, Lawrence Berkeley National Laboratory, Berkeley, California, USA; 3 Centre for Computational Medicine Hospital for Sick Children, Toronto, Ontario, Canada; 4 Department of Computer Science, University of Toronto, Toronto, Ontario, Canada; 5 Medical Genetics Branch, National Human Genome Research Institute, Bethesda, Maryland, USA; 6 Skarnes Faculty group, Wellcome Trust Sanger Institute, Hinxton, UK; 7 Institute for Medical Genetics and Human Genetics, Charité-Universitätsmedizin Berlin, Berlin, Germany; 8 Library; and Department of Medical Informatics and Epidemiology, Oregon Health & Science University, Portland, Oregon, USA

**Keywords:** exome sequencing, model organisms, phenotype, semantic comparison, undiagnosed diseases

## Abstract

**Purpose:**

Medical diagnosis and molecular or biochemical confirmation typically rely on the knowledge of the clinician. Although this is very difficult in extremely rare diseases, we hypothesized that the recording of patient phenotypes in Human Phenotype Ontology (HPO) terms and computationally ranking putative disease-associated sequence variants improves diagnosis, particularly for patients with atypical clinical profiles.

*Genet Med* **18** 6, 608–617.

**Methods:**

Using simulated exomes and the National Institutes of Health Undiagnosed Diseases Program (UDP) patient cohort and associated exome sequence, we tested our hypothesis using Exomiser. Exomiser ranks candidate variants based on patient phenotype similarity to (i) known disease–gene phenotypes, (ii) model organism phenotypes of candidate orthologs, and (iii) phenotypes of protein–protein association neighbors.

*Genet Med* **18** 6, 608–617.

**Results:**

Benchmarking showed Exomiser ranked the causal variant as the top hit in 97% of known disease–gene associations and ranked the correct seeded variant in up to 87% when detectable disease–gene associations were unavailable. Using UDP data, Exomiser ranked the causative variant(s) within the top 10 variants for 11 previously diagnosed variants and achieved a diagnosis for 4 of 23 cases undiagnosed by clinical evaluation.

*Genet Med* **18** 6, 608–617.

**Conclusion:**

Structured phenotyping of patients and computational analysis are effective adjuncts for diagnosing patients with genetic disorders.

*Genet Med* **18** 6, 608–617.

## Introduction

The National Institutes of Health (NIH) established the Undiagnosed Diseases Program (UDP) to provide explanations to individuals with elusive disorders and to improve understanding of rare and common disorders. Of the individuals or families admitted, ~15% are diagnosed with a known condition based on clinical evaluation.^1,2^ For those remaining without a diagnosis, the NIH UDP screens for potential genetic etiologies. The diagnoses have included known disorders that were previously unrecognized, atypical presentations of known disorders, combinations of several disorders, and completely novel diseases.^2,3^

Exome sequencing identifies thousands of exomic variations relative to the human reference sequence, and this often precludes generation of a tractable number of diagnostic hypotheses. This problem remains after filtering for predicted deleteriousness, conservation, and Mendelian modes of inheritance.^4^ Many variants may even be true loss-of-function mutations tolerated in the average human genome.^5,6^ Also, because the UDP often has DNA from only the proband or a few family members, or sometimes a few unrelated patients for each disease being studied, traditional linkage and cohort analyses are not possible.

Encoding patient phenotypes using an ontology such as Human Phenotype Ontology (HPO) assists computational ranking and identification of causal variants.^7,8,9,10^ However, because only a small portion of human coding genes (~35% by our estimation) have been associated with disease, candidate variations in genes with unknown function or phenotypic consequences are difficult to evaluate. To address this, the first version of Exomiser prioritized causative variants using semantic comparisons of encoded mutant mouse phenotypes against the HPO-encoded patient phenotypes as well as variant frequency, pathogenicity, and inheritance models.^10^ Despite improving variant identification, Exomiser was still biased to identify causal variants in genes for which their orthologs had a phenotype annotated. We therefore hypothesized that querying human, mouse, and zebrafish mutant phenotypes together and the phenotypes associated with mutations of interacting proteins can improve Exomiser function.

To test this hypothesis, we recorded structured phenotype descriptions utilizing HPO and performed Exomiser analysis of sequence variants from 32 UDP families (9 diagnosed and 23 undiagnosed). This showed that ontology-based phenotype analysis, cross-species integration, and protein–protein association walking aid the prioritization of candidate variants and lead to more efficient diagnoses as well as to the generation of plausible novel disease–gene hypotheses.

## Materials and Methods

### Human subjects

Patients or their guardians gave written informed consent to protocol 76-HG-0238, approved by the Institutional Review Board of the National Human Genome Research Institute. We analyzed 9 families diagnosed by the UDP clinicians and 23 families whose affected members remained undiagnosed when we began this investigation.

### Patient phenotype documentation and curation

As culled from medical records, the patient phenotype was recorded using the PhenoTips (http://phenotips.org)^11^ tool using HPO, curated by members of the Monarch Initiative according to qualitative guidelines and quantitative graph-based metrics (http://monarchinitiative.org/page/services), and approved by the attending physician.^12^ Positive HPO terms were exported from PhenoTips and used for Exomiser analysis (**Supplementary Table S1** online); negative and onset of disease terms are not currently utilized by the algorithm. For families with phenotype data from multiple affected individuals, we used the intersection of the HPO terms. All patient data (phenotypes and exomes) were uploaded to the PhenomeCentral patient-matching portal (https://phenomecentral.org) to identify patients with similar phenotypes and candidate genetic mechanisms.

### Exome sequencing

Exome sequencing and analysis were performed as described.^13,14,15,16^ Further details are provided in the **Supplementary Methods** online.

### Filtration and evaluation of exome variants

After filtering the variants for rarity, segregation, and deleteriousness as described in the **Supplementary Methods** online (**Supplementary Figure S1** online), the variants were submitted to Exomiser for ranking. Note the variants are filtered separately under compound heterozygous, homozygous recessive, de novo dominant, and X-linked inheritance models, and the output from these four models is combined before ranking by Exomiser. Following ranking of the variants by Exomiser, we reviewed the quality of alignment and genotype of the highest-ranked variants as described in the **Supplementary Methods** online.

### Ranking of variants using Exomiser

Using the data sources described in the **Supplementary Methods** online, Exomiser assesses variant candidacy by allele frequency, predicted pathogenicity, and inheritance model, as well as the likelihood of a mutation in the gene causing the disease based on the similarity of a patient's phenotype to known gene-associated phenotypes in human, mouse, or zebrafish. Additionally, for candidate genes that have no terms that match the patient, the proximity of a candidate gene to another phenotypically similar gene via protein–protein associations is leveraged.

Genes were ranked for candidacy by Exomiser's variant and phenotypic relevance score. The optimal method of combining these scores was generated by logistic regression on a training set of 10,000 Human Gene Mutation Database (HGMD) disease variants and 10,000 benign variants from the 1000 Genomes Project. The Weka data mining suite was used to run the logistic classifier on files containing phenotype and variant scores for equal amounts of disease and benign variants. Ten-fold cross-validation was used to train and test the model (91.5% of variants were correctly classified in the cross-validation, *κ* = 0.8296) and average parameters from the cross-validation runs were used to generate the final model^17^:


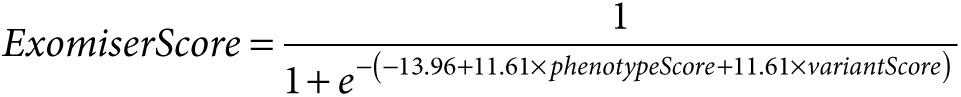


### Variant score

Exomiser's variant score is a measure of how rare and pathogenic the variant is. A variant frequency score between 0 and 1 was calculated as previously optimized and described^10^:

Frequency score = max (0,1–0.13533*e*^100*f*^), where f is the maximal minor allele frequency seen in the 1000 Genomes or Exome Sequencing Project (ESP) between 0 and 1.

Predicted pathogenicity scores for missense variants (0 [benign] and 1 [pathogenic]) were obtained by taking the maximum score for the variant from Polyphen2, MutationTaster, or 1 minus the SIFT score. For other classes of variants, the pathogenicity scores were assigned as described.^10^ The final variant score was the product of the pathogenicity and frequency scores. When no data were available from all three algorithms, a default variant score of 0.6 was used. For compound heterozygous pairs, the final score was the average of the final variant score for the two highest scoring alleles for a gene.

### Phenotypic relevance score

**Figure 1:**
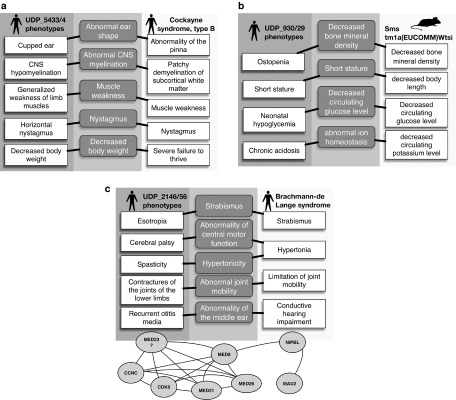
**Semantic phenotype matching**. For each variant that passed the frequency and Mendelian inheritance filters, the patient's Human Phenotype Ontology terms were compared to all human diseases associated with the gene containing the variant in OMIM or Orphanet, (**a**) as well as any phenotypes associated with orthologs of the gene in mice or zebrafish (**b**). If there was no phenotypic match between the patient and any phenotypes associated with the gene, then the patient's phenotype was compared with phenotypes associated with nearby genes in the protein–protein association network (**c**). When calculating the phenotypic score, the network considered the similarity to the patient phenotype and proximity of the matching gene to the gene in which the patient had a mutation.

Similarities between a patient's HPO phenotypic profile and known gene–phenotype annotations in human, mouse, and zebrafish were scored by semantic similarity.^18^ This approach allows related, but nonexact, phenotypes to be detected and is used to generate a similarity score based on how alike the terms are and how specific the match is. Exomiser generates a phenotype score between 0 and 1 by pairwise comparisons between the HPO-annotated disease or patient and any annotations present for the human (**Figure 1a**), mouse (**Figure 1b**), or zebrafish genes being assessed.^18^.

The Exomiser algorithm also compares patient phenotypes to the phenotypes of nearby genes in a protein–protein association network. The network was defined by high-confidence (>0.7) interactions from STRING (Search Tool for the Retrieval of Interacting Genes/Proteins), version 9.05. The high-confidence interactions include direct (physical) and indirect (functional) protein–protein interactions, as well as associations transferred by orthology from other species or obtained through text mining^19^ (**Figure 1c**). To score proximity to a candidate gene, Exomiser used a random-walk method previously optimized for candidate gene identification^20,21^: starting from the candidate gene, the walker moved randomly around the network with a restart probability of 0.7, and the final output was a probability vector summing to 1 and giving scores between 0 and 1 depending on the proximity to the candidate gene. The proximity score for that gene was used to weight each phenotypic relevance score. After scoring all candidate genes for variants obtained from a filtered exome, the network analysis scores are scaled to 0 to 0.6 based on their rank to give the final phenotypic relevance score. The maximum value of 0.6 was based on optimizations in the known disease variant simulations. The final phenotypic relevance score is the maximum score of the comparisons between the disease/patient phenotypes and the human, mouse, or fish annotations for the candidate gene or its neighbors in the interactome.

### Code availability

Exomiser can be used online or downloaded as a command line tool for local use (https://www.sanger.ac.uk/resources/software/exomiser or ftp://ftp.sanger.ac.uk/pub/resources/software/exomiser/downloads/exomiser).

### Exomiser benchmarking

Benchmarking experiments for Exomiser were performed using 10,000 simulated rare disease exomes based on 28,516 known disease-causing mutations from the HGMD database (http://www.hgmd.org, accessed 1 March 2012, RRID:nif-0000-10459) and 1092 whole-exome variant call format (VCF) ﬁles from the 1000 Genomes Project (http://www.1000genomes.org/data, 2 May 2013 release). Ten thousand exomes were chosen to effectively cover the range of known disease mutations and phenotypes. For autosomal dominant diseases, one heterozygous mutation was added; for autosomal recessive diseases, either one homozygous mutation or two heterozygous mutations were added to the 1000 Genomes VCF ﬁle. For these experiments, the phenotypic (HPO) annotations for the corresponding disease in OMIM were taken on 1 August 2014 from the annotation files of the HPO team (http://compbio.charite.de/hudson/job/hpo.annotations/lastStableBuild).

These simulated exomes were run through the default settings of Exomiser, applying a 1% minor allele frequency cutoff and either no inheritance model or the known autosomal dominant or recessive model. To measure the ability of Exomiser to detect known disease–gene associations, we repeated the analysis with incomplete (maximum of five HPO annotations), noisy (two random HPO terms added), and imprecise (two of the HPO annotations replaced by the more general parent terms in the ontology) annotations. To measure Exomiser's performance in identifying novel disease–gene associations, we repeated these runs but excluded from the prioritization algorithm the human disease–gene association being tested.

We used allele frequency data from the ESP (http://evs.gs.washington.edu/EVS/) and the 1000 Genomes Project while running Exomiser on these simulated exomes. These analyses were performed excluding the 1000 Genomes Project data as well, because using population frequency data from the same source as our simulated exomes provides unrealistically effective frequency filtration. To assess our performance, we measured how often the seeded HGMD causative gene and variant(s) were ranked first. An ordinal ranking method was used to resolve equivalently scored genes by assigning a unique rank to each of the ties; i.e., we sorted the equally scored genes alphabetically and assigned rank.

## Results

### Evaluation of a phenotype-aware variant prioritization algorithm

To assess the performance of the enhanced Exomiser algorithm, we performed a benchmarking analysis on 10,000 simulated exomes (**Table 1** and **Supplementary Figure S2** online) seeded with HGMD variants according to various knowledge conditions and inheritance models (as described in Materials and Methods). Filtering to remove variants with a minor allele frequency above 1% was performed using either ESP and 1000 Genome Project data or ESP data alone. The clinical phenotypes associated with the HGMD variant were specified as HPO terms.

Depending on the inheritance model and frequency data, Exomiser ranked the causative variant as the top hit for 96–97% of the simulated exomes. For imperfect phenotypic profiles (as described in Materials and Methods), Exomiser ranked the causative variant as the top hit for 94–96% when using all available frequency data and for 90–92% when using only ESP frequency data. In contrast, using a variant-based approach alone, the seeded variant was the top hit for only 20–77% of the exomes using all available frequency data and was only 1–10% when using only ESP frequency data. For known disease–gene associations, it may have been expected that the causative gene would be ranked by phenotype matching as the top hit 100% of the time. In reality, some of the disease annotations were not specific enough to distinguish the seeded variants in the disease-associated gene from variants in other genes.

Having shown that Exomiser detects known disease–gene associations regardless of the inheritance model and with incomplete variant frequency data or phenotype, we tested its detection of novel disease–gene associations through deduction from phenotypically similar diseases, mouse and fish models, or protein-protein association (PPA) data. To do this, we removed the disease–gene association being tested from the underlying dataset. Exomiser ranked the causative variant as the top hit for 74–87% when using all available frequency data and for 52–64% when using only ESP frequency data.

**Figure 2:**
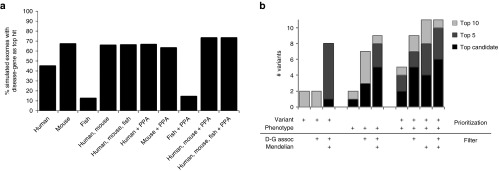
**Benchmarking of Exomiser prioritization**. (**a**) The contribution of human, mouse, and zebrafish phenotypes along with protein–protein association data to novel association discovery is shown for each alone and the various combinations. HGMD mutations were added to unaffected 1000 Genomes Project exomes and run through Exomiser under conditions where the known disease–gene association was removed from the database for each run to simulate novel discovery. Bars show percentage of exomes in which the true variant was prioritized as the top hit. Results shown are after filtering to remove common (>1% minor allele frequency by Exome Sequencing Project data), synonymous, and noncoding variants. (**b**) Performance on previously diagnosed Undiagnosed Diseases Program disease variants. Shown are rankings of 11 previously diagnosed variants from nine solved families when analyzed under different conditions as indicated in the table below the chart: *prioritization* was based on the *variant* score alone (allele frequency and pathogenicity) and/or in combination with the *phenotype* score, and *filtering* was run with and without inclusion of pedigree-defined *Mendelian* filtering and inclusion of the disease–gene *(D-G)* association. Bars show how many of the 11 previously diagnosed variants were on the list of the top 1, 5, or 10 candidate variants. The Exomiser scores are reflected in the last two columns, which incorporate variant and phenotype. The best performance is observed with inclusion of a known Mendelian inheritance model; all 11 variants were in the top 5 or 10, with or without prior knowledge of disease–gene associations, respectively.

To assess further the contribution of the human, mouse, and zebrafish phenotypes as well as PPA data, we used various combinations of each in the prioritization algorithm (**Figure 2a** and **Supplementary Table S2** online). For known associations, the main contributor to performance was the matching of the clinical phenotypes to the known human disease phenotypes. For novel associations, looking at the individual species phenotype performances alone, mouse performed the best, followed by human, and then zebrafish. Combining human and mouse phenotype data with PPA data led to a significant performance increase relative to human or mouse alone. Combining human and fish phenotype data also increased performance relative to either alone. When we assessed the 45.4% of samples for which human alone was able to prioritize the variant as the top hit, we found that only 0.5% of these were no longer the top hit when mouse and fish data were added; i.e., addition of the model organism data does not lead to significant loss of true positives and increases the chances of detecting the causative variant. When assessing the performance of mouse or fish alone (68 and 13%, respectively), it should be considered that only 91 and 32% of the analyzed samples had phenotype data for the disease gene from mouse or fish, respectively. If one takes into account only samples with model phenotype data, then 75 and 41% of mouse or fish had the causative variant as the top hit when used as the only model data. To understand the contribution by the fish data that were added in this new version of Exomiser, we analyzed the 15% of simulated exomes for which using fish phenotypes plus PPA data ranked the causative variant as the top hit and found that in this dataset the human or mouse data had the same gene as the top hit as the fish in 78% of cases, with false positives from human and mouse outscoring the fish hit in the other 22%. Hence, the fish data currently serve as supporting evidence. Overall the combination of phenotype data from multiple species with PPA data improves detection of novel associations from 45% for a human phenotype–only approach to 74% with the combined approach.

### Benchmarking on previously diagnosed families

We next assessed the performance of Exomiser when using the structured phenotypic descriptions and filtered VCF files for nine previously diagnosed families representing 11 disease–gene associations (**Supplementary Table S5** online and **Figure 2b**). As detailed in Materials and Methods, the filtered VCF files contain the combined output after filtering for compound heterozygous, homozygous recessive, de novo dominant, and X-linked variants; therefore, Exomiser ranking is performed using this combined total; i.e., ranks are out of an average of 54 genes and the inheritance pattern of the diagnosed variant does not influence performance. The combined Exomiser score ranked each of the 11 disease-causing variants in the top seven hits of their respective lists, and 6 of 11 ranked first. Five of the matches were based on known human disease phenotypes, one was via phenotypic similarity to the mouse model, and the other five were based on protein–protein association matches to other human diseases. For example, the *AARS2* variants in UDP_4306 matched via the protein–protein association analysis to *EARS2* and the disease combined oxidative phosphorylation deficiency 12 (OMIM:614924).

To evaluate the effectiveness of the combined variant and phenotype score, we compared the Exomiser ranking to each component score. In the respective VCFs, the variant score ranked 8 of 11 disease-associated variants among the top 10 and 1 was ranked first, whereas the phenotype score alone ranked 9 of 11 disease-associated variants among the top 10 and 5 were ranked first.

To simulate prioritization of these variants as previously undescribed diseases, we removed the known disease–gene association from the Exomiser database. Exomiser prioritized all disease-associated variants in the top 10 of their respective lists (**Figure 2b**).

Using the parental and sibling DNA sequence facilitates the identification of sequence variants segregating with disease; however, these data often are not collected or are unavailable. The inability to filter variants for segregation with disease leaves a much larger list of plausible variants and increases the difficulty of prioritizing candidate variants. To test Exomiser in this scenario, we compared the outcome for known causative variants when parental and unaffected sibling exome data were not used. Exomiser prioritized 9 of the 11 variants within the top 10 candidates of each VCF file; of these, 5 ranked first (**Figure 2b**).

### Exomiser prioritizes variants of undiagnosed individuals to aid diagnoses of known diseases

We next investigated whether Exomiser could prioritize variants and thereby suggest a diagnosis for individuals in the UDP cohort whose conditions had eluded clinical elucidation. Using VCF files filtered for Mendelian segregation and allele frequency, Exomiser prioritized variants such that the clinical team made diagnoses for two individuals in a cohort of 21 (**Table 2**). Specifically, the diagnosis of UDP_6392 with Kufor-Rakeb syndrome (OMIM:606693) was based on clinical correlation with the biallelic *ATP13A2* mutation resulting in *ATP13A2* ranking as the top candidate of 107 genes. In addition, UDP_4964 was diagnosed with HARP syndrome (OMIM:607236) due to compound heterozygous *PANK2* mutations, with *PANK2* ranking first of 221 genes.

### Exomiser prioritizes variants with previously unknown disease–gene associations

To test if Exomiser could prioritize variants to allow molecular characterization of a new disease, we reviewed the results for the remaining 19 of the 21 families in the undiagnosed cohort. Exomiser analysis provided viable candidates for 17 (**Supplementary Table S3** online); most of the candidates were based on strong variant scores and indirect phenotype evidence via protein–protein associations. UDP_2058 and UDP_3478 had no variants meeting our criteria for a deleterious mutation and with a gene frequency of <2% in the UDP population. For patients in two families affected by York platelet syndrome, a disorder of then unknown molecular cause, Exomiser prioritized mutations in *STIM1* (**Table 2**). Subsequent study of two additional families with York platelet syndrome confirmed association with mutations in *STIM1.*^22^ PhenomeCentral independently identified two of these patients (UDP_2542 and UDP_2543) as reciprocal best phenotype matches, with *STIM1* highlighted as the best-scoring shared genetic mechanism.

## Discussion

We show that computational comparison of patient phenotype to human, mouse, and zebrafish combined with PPA guilt-by-association phenotype data improves computational prioritization of exome sequencing variants. This new version of Exomiser was able to rank known disease-associated variants first in up to 97% of the HGMD spiked exomes, which is a 30% improvement over the original version that used mouse phenotypes alone.^10^ In the clinical setting of the NIH UDP, Exomiser not only prioritized known disease-causing mutations with or without previous knowledge of these associations or the inheritance model but also, when tested using data from patients undiagnosed by clinical evaluation, ranked a diagnostic variant within the top 10 for 4 of 23 cases. This latter observation suggests that this computational approach at least partially overcomes human bias to rank diagnostic sequence variants more efficiently. It is difficult to predict from the benchmarking presented here exactly how much Exomiser may be expected to increase the diagnostic rates from the 25–30% typically seen in exome sequencing projects. Computationally identifying a strong candidate is not the same as making a clinical diagnosis, particularly for variants found in genes not yet associated with human disease, and projecting performance from the 32 UDP patients we analyzed with their particular complexities may not be valid for all projects. However, we expect that the approach we have presented here would be useful for any group investigating clinical diagnostics through exome sequencing.

Our observations also suggest that the difficulties faced in variant analysis due to the absence of parental genotype data can be overcome at least partially by deep structured phenotyping and the use of Exomiser. In the absence of parental sequence data, Exomiser ranked diagnostic variants for 9 of 11 UDP patients within the top 10 of each VCF file. Inclusion of Mendelian segregation provided the most assistance in the context of weaker phenotype matches; this emphasizes the need for thorough structured phenotyping. To ensure such a thorough phenotype, we implemented both manual and computational quality assurance procedures to indicate the phenotype quality with respect to breadth and depth by comparison of all phenotype profiles for human diseases and model organisms (see Materials and Methods).

Because atypical and difficult to diagnose conditions do occasionally arise from co-occurrence of genetic disorders, a test of the strength of a tool such as Exomiser is its ability to prioritize variants from each of the co-occurring conditions. When siblings UDP_606 and UDP_608 were analyzed individually in trio analyses, Exomiser was able to rank the *RAI1* and *PCK1* mutations as the first and third candidates for UDP_606 and *PCK1* and *GRIN2B* mutations as the second and fifth candidates for UDP_608 (**Supplementary Table S4** online).^2^ Therefore, given an appropriate experimental design, Exomiser can effectively rank diagnostic variants of multigenic disorders.

As may be expected, Exomiser's performance is challenged when the variant is in a gene not previously implicated in the disease. We observed a 10–41% reduction in Exomiser's ability to rank known disease-associated variants as the top candidate when we simulated discovery of these variants through removal of these disease–gene associations in the benchmarking experiments. There was a similar reduction in ranking for diagnostic variants within the UDP cohort. Exomiser is nonetheless a valuable tool for generating hypotheses for undiagnosed disease because (i) the relevant variants remain highly ranked in a reasonably sized candidate list, (ii) known disease–gene associations are rapidly prioritized or dismissed, and (iii) evidence behind each prediction is presented for evaluation.

Our benchmarking revealed that, for known associations, the matching of patient phenotypes to known human diseases was the main contributor to performance. For novel associations, human, mouse, and PPA data contributed additively to performance. In the vast majority of the simulated cases and all the positive controls we investigated, the addition of more species did not have an effect on the ability of Exomiser to identify the causative variant. In the 0.5% of simulated cases where the causative variant identified using human data was replaced by a false-positive model organism phenotype match, this was generally due to the human disease phenotypes not being very specific. Using model organism phenotype data alone was effective, with 75% (mouse) and 41% (fish) of exomes having the causative variant as the top hit when restricting to samples with existing animal models, or 68% (mouse) and 13% (fish) for all samples. Investigations of the exomes where zebrafish alone could rank the causative variant as the top hit revealed that in almost 80% of cases human and/or mouse matches already ranked it first. For nearly all the other cases, false-positive human and/or mouse matches outscored zebrafish. However, we would argue that it is currently important to include zebrafish in Exomiser analyses as supporting evidence, and in the Exomiser output we do present evidence from all species; e.g., equivalent scoring human/mouse variants can be distinguished if one has good evidence from zebrafish as well. Future versions of the algorithm will formalize this by scoring the combined evidence from all organisms rather than the current strategy of taking the best score. The inclusion of zebrafish data also has provided valuable insight regarding how to best include additional species in future Exomiser versions. We have observed that there is an approximately twofold increase in phenotypic coverage of human coding genes in model organism orthologs (mouse, rat, zebrafish, fruitfly, and worm) over human genes alone (ClinVar, genome-wide association studies, and OMIM). It will therefore be important to leverage as many species as possible to have the deepest genotype–phenotype coverage of the human genome.

The diagnostic capability of the methodology considered herein can and should improve as the mutant model organism databases accumulate more data and as improvements are made to the phenotype comparison algorithm. Initiatives such as the International Mouse Phenotyping Consortium^23^ and the Zebrafish Mutation Project^24^ will greatly expand the available knowledge about gene–phenotype relationships. Also, incorporation of additional species' phenotype data into Exomiser will improve its performance. Another improvement will come with the use of absent phenotypes. For example, the lung phenotype of UDP_2700 mapped well to Fraser syndrome but the absence of syndactyly and severe neurological symptoms was not considered, and thus Fraser syndrome ranked inappropriately high. These improvements are currently being implemented in the OWLsim algorithm (http://www.owlsim.org)^25^ and will be incorporated into the next version of Exomiser.

Some patients likely have genetic disorders unsolvable by exome sequencing and Exomiser alone. Besides the possibility that the initial assumption of a germline genetic basis for the disease might be invalid, exome data only cover 2% of the genome and are insensitive to certain types of mutations, including copy number variations and trinucleotide repeats. At present, the UDP uses single-nucleotide polymorphism chips as part of the standard analysis for patients. Adding these data to our analysis would have identified the intragenic *MEGF10* deletion in UDP_2473.^26^ We have also previously shown that phenotypic semantic similarity analyses on copy-number variations help explain the genetic contribution to patient phenotypes.^27^ Finally, we will need to adapt future versions of Exomiser to assess the significantly greater number of variations identified by whole-genome sequencing data and develop effective methods of prioritizing the mainly intronic and intergenic variations that these data provide.

Other approaches have also utilized phenotype data and predicted deleteriousness to prioritize exomic variants. PhenoVar is an exome analysis tool that prioritizes variants based on patient phenotype similarity to HPO terms associated with OMIM disease,^9^ and, as we have also observed, this comparison to known human phenotypes is frequently all that is necessary.^28^ eXtasy also incorporates multiple lines of evidence, including clinical phenotypes to predict the deleteriousness of nonsynonymous mutations.^29^ PHEVOR compares patients and diseases by using an ontology propagation approach using the Mammalian Phenotype ontology, HPO, and Gene Ontology terms.^8^ Although the PHEVOR method increases the number of genes that have phenotypic information and the amount of phenotypic data associated with a gene, it still requires that phenotype data are available for the gene containing the variant. Phen-Gen utilizes phenotype matching and a random-walking algorithm, but without taking advantage of model organism data;^30^ its benchmarking, using the same strategy with 1000 Genome Project exomes, spiked HGMD variants and ESP and 1000 Genomes Project frequency data, revealed a 13–58% improvement over all existing prediction methods, including eXtasy, PHEVOR, and the previous version of Exomiser. The performance of the current version of Exomiser with 97% for both autosomal dominant and autosomal recessive disorders compares favorably with Phen-Gen's performance of 92 and 96% for dominant and recessive disorders, respectively. When using the same masking strategy to detect novel associations for autosomal dominant and recessive disorders, Phen-Gen performs at 56 and 89% compared to 79 and 87% for the current version of Exomiser. Compared to the prior version of Exomiser, this represents an improvement in detection of novel associations by 4–13% and of known associations by 16–31%. This suggests that Exomiser is currently the optimal solution to detecting known and novel disease variants.

Exomiser takes advantage of cross-species phenotype data using semantic bridging ontologies,^31^ and this capability produces the increased performance we observed in our benchmarking over other exome prioritization tools. This ability to stretch phenotypic coverage by leveraging cross-species genotype–phenotype data is particularly important in programs like the UDP, where the patients have unusual phenotypes that may be caused by variation in an as yet unstudied gene. We have already shown the utility of expanding the phenotypic matches by our ability to prioritize *AARS2* variants in UDP_4306, *GRIN2B* variants in UDP_608, *MED23* variants in UDP_2146/2156, and *SMS* variants in UDP_930/929 (**Table 2**). Interestingly, the *SMS* match is to the *Sms* mouse data generated by the International Mouse Phenotyping Consortium; no other *Sms* mutant mouse descriptions reported the glucose and potassium abnormalities that were critical to identifying this variant. We therefore speculate that expansion of the phenotype coverage in the International Mouse Phenotyping Consortium database over the next few years will enable many more discoveries like this. The inclusion of the protein–protein association data was also of particular importance for the UDP patients, where 5 of the 11 disease variants found in previously diagnosed families were detected by Exomiser via this link. In all cases, these patients were diagnosed with diseases known to be associated with the mutated gene. However, the patient's phenotypic profile was so atypical that only weak matches at best were detected for these diseases. Instead, Exomiser detected strong phenotypic matches to protein–protein association neighbors of the mutated gene.

In summary, combining Mendelian segregation filters, population frequency, and variant predicted deleteriousness with structured phenotype data from humans and model organisms improves diagnostic efficiency and effectively identifies and prioritizes exome variants associated with known and new diseases. Comparing patient phenotype to human disease phenotype data is particularly useful to identify known human diseases, whereas for those human genes currently unassociated with disease, increasing phenotypic coverage by using model organism and protein–protein association data allows generation of hypotheses for the genetic basis of a disease.

## Disclosure

The authors declare no conflict of interest.

## Figures and Tables

**Table 1:**
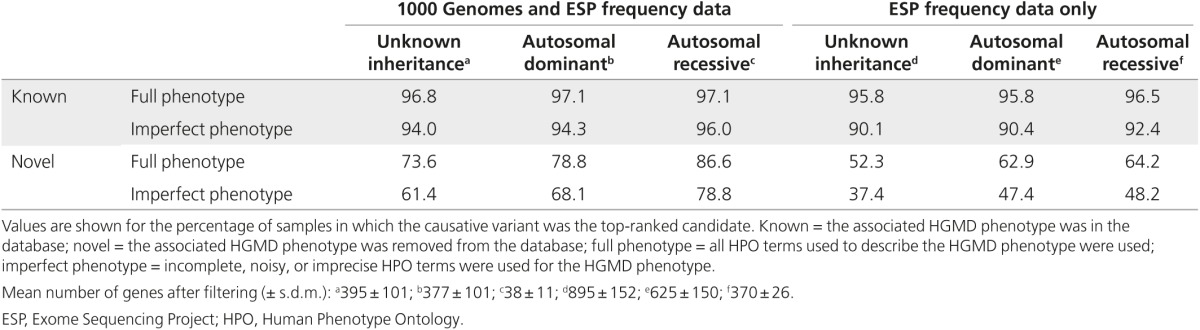
Benchmarking of Exomiser on simulated samples produced by adding known disease variants from HGMD to unaffected 1000 Genomes Project exomes

**Table 2:**
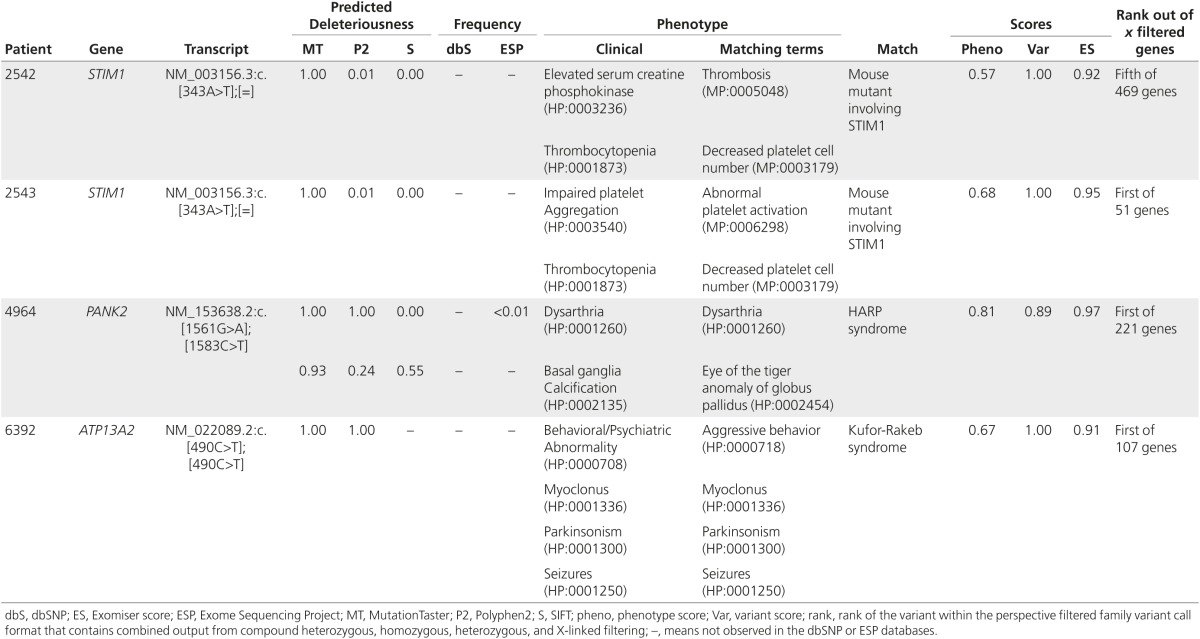
Exomiser ranking of variants leading to new diagnoses
