# Supplementary material for: Computational evaluation of exome sequence data using human and model organism phenotypes improves diagnostic efficiency
Source: Genet Med. 2015 Nov 12;18(6):608–17. doi: 10.1038/gim.2015.137 (PMC4916229; doi:10.1038/gim.2015.137)
Supplement: Supplementary Figure S1 [file gim2015137x1.pdf]

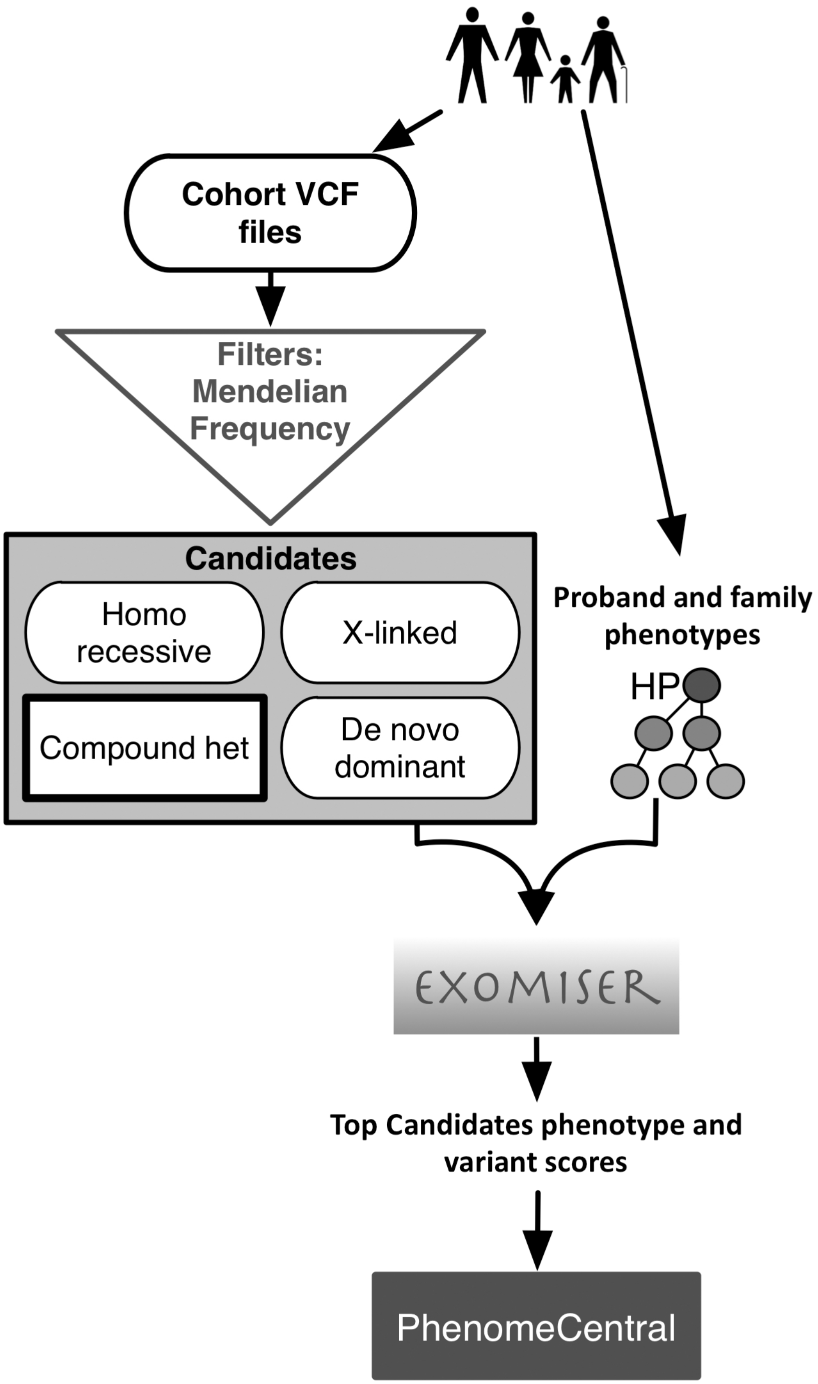

Figure S1. Analysis pipeline overview. Exome sequencing was performed on the proband and, where available, parents and siblings. Each person's phenotype was recorded using Human Phenotype Ontology terms using PhenoTips. The VCF files were filtered based on minor allele frequency, and Mendelian segregation patterns were used as inputs to Exomiser, along with the HPO terms for the patient. Exomiser prioritized the variants according to pathogenicity, frequency, phenotypic similarity within and across species, and protein-protein association networks. Compound heterozygous variants were assessed as a single candidate and the variant scores of the individual heterozygous variants were aver aged to generate the candidate variant score. The quality of the top candidates from Exomiser was verified by manual inspection of the alignment and genotype calls, which were cross referenced with the PhenomeCentral database.
