# Supplementary material for: Computational evaluation of exome sequence data using human and model organism phenotypes improves diagnostic efficiency
Source: Genet Med. 2015 Nov 12;18(6):608–17. doi: 10.1038/gim.2015.137 (PMC4916229; doi:10.1038/gim.2015.137)
Supplement: Supplementary Figure S2 [file gim2015137x2.pdf]

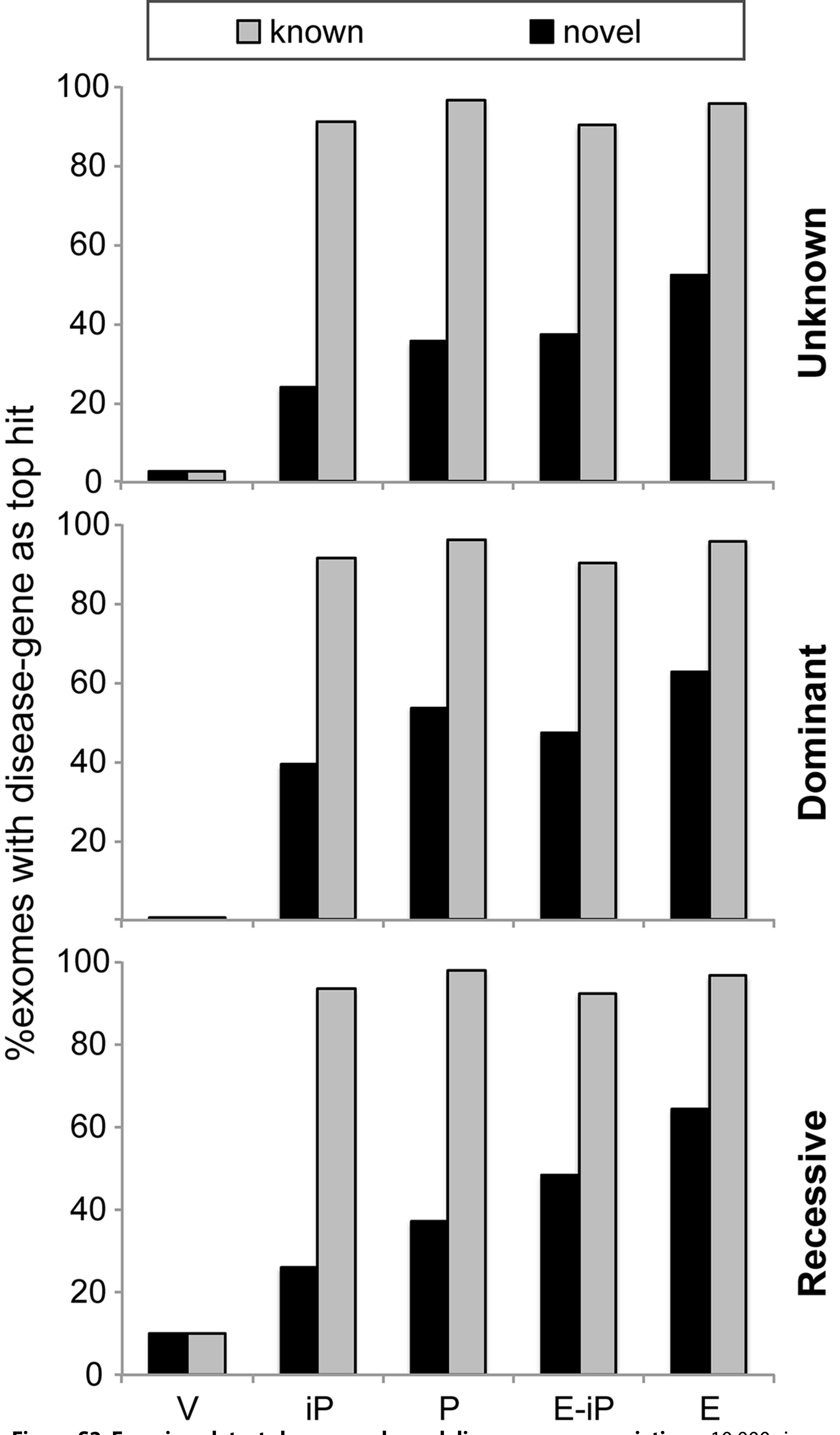

Figure S2. Exomiser detects known and novel disease-gene associations. 10 000 simulated WES datasets were created by injecting single disease-causing variants into exomes from Phase I 1000 Genomes Project (1000G) then analyzed to compare performance of Exomiser under various conditions. Bars show percentage of exomes in which the true variant was assigned as the best match based on prioritization and analysis scored as follows: Variant (V) score alone used allele frequency from the ESP and pathogenicity data; imperfect phenotype (iP) used set of gene-phenotype associations in which some of the anno tated phenotypes of the tested disease were removed, others made more generic, and random annotations added; all phenotype (P) included all phenotypes for the tested disease; Exomiser (E) score used a combination of variant, phenotype, and disease-gene associations; Exomiser with imperfect phenotype (E-iP). Both sets of scoring methods were assessed with (known, grey bar) or without (novel, black bars) including the known disease-gene associations, to simulate a situation for when a causative gene is novel. Results shown are after filtering by removal of common (> 1% minor allele frequency), syno mous, and non-coding variants, followed by application of an inheritance model for autosomal dominant (AD), recessive (AR), or unknown. Mean gene counts before filtering was 8388 +/-882 (s.d.m.); mean post-filtering gene count for ESP was 895 +/- 152 (unknown inheritance); 625 +/- 150 (AD inheritance); 370 +/- 26 (AR inheritance).
