# Supplementary material for: Computational evaluation of exome sequence data using human and model organism phenotypes improves diagnostic efficiency
Source: Genet Med. 2015 Nov 12;18(6):608–17. doi: 10.1038/gim.2015.137 (PMC4916229; doi:10.1038/gim.2015.137)
Supplement: Supplementary Table S1 [file gim2015137x3.doc]

**Table S1. Full list of positive HPO terms for each participating patient and family**.

| Patient | HPID | HPO term |
| --- | --- | --- |
| 579/577 | HP:0000156 | High palate |
|  | HP:0000280 | Coarse facial features |
|  | HP:0000425 | Depressed nasal bridge |
|  | HP:0000505 | Visual impairment |
|  | HP:0001303 | Seizures |
|  | HP:0001622 | Premature birth |
|  | HP:0002133 | Status epilepticus |
|  | HP:0002197 | Generalized seizures |
|  | HP:0007202 | Generalized myoclonic seizures |
|  | HP:0007930 | Prominent epicanthal folds |
|  | HP:0010804 | Tented upper lip vermilion |
|  | HP:0011344 | Severe global developmental delay |
|  | HP:0012448 | Delayed myelination |
| 579 | HP:0011344 | Severe global developmental delay |
|  | HP:0002283 | Global brain atrophy |
|  | HP:0012448 | Delayed myelination |
|  | HP:0011471 | Hypoplasia of the brainstem |
|  | HP:0002197 | Generalized seizures |
|  | HP:0002319 | Hypoplasia of the corpus callosum |
|  | HP:0007202 | Generalized myoclonic seizures |
|  | HP:0001303 | Seizures |
|  | HP:0002133 | Status epilepticus |
|  | HP:0001629 | Ventricular septal defect |
|  | HP:0000318 | Narrow face |
|  | HP:0000537 | Epicanthus inversus |
|  | HP:0000156 | High palate |
|  | HP:0010804 | Tented upper lip vermilion |
|  | HP:0010759 | Premaxillary Prominence |
|  | HP:0007930 | Prominent epicanthal folds |
|  | HP:0000621 | Entropion |
|  | HP:0000280 | Coarse facial features |
|  | HP:0000425 | Depressed nasal bridge |
|  | HP:0001845 | Overlapping toe |
|  | HP:0100667 | Brachydactyly syndrome |
|  | HP:0001169 | Broad palm |
|  | HP:0011471 | Gastrostomy tube feeding in infancy |
|  | HP:0000505 | Visual impairment |
|  | HP:0001622 | Premature birth |
|  | HP:0008936 | Muscular hypotonia of the trunk |
|  | HP:0011471 | Cerebellar hypoplasia |
|  | HP:0012389 | Appendicular hypotonia |
| 577 | HP:0011344 | Severe global developmental delay |
|  | HP:0002197 | Generalized seizures |
|  | HP:0007202 | Generalized myoclonic seizures |
|  | HP:0001303 | Seizures |
|  | HP:0002133 | Status epilepticus |
|  | HP:0001631 | Defect in the atrial septum |
|  | HP:0000156 | High palate |
|  | HP:0010804 | Tented upper lip vermilion |
|  | HP:0000280 | Coarse facial features |
|  | HP:0000425 | Depressed nasal bridge |
|  | HP:0007930 | Prominent epicanthal folds |
|  | HP:0000023 | Inguinal hernia |
|  | HP:0001252 | Muscular hypotonia |
|  | HP:0000505 | Visual impairment |
|  | HP:0012382 | Left-to-right shunt |
|  | HP:0002019 | Constipation |
|  | HP:0012448 | Delayed myelination |
|  | HP:0001622 | Premature birth |
| 606/608 | HP:0001251 | Ataxia |
|  | HP:0001263 | Global developmental delay |
|  | HP:0002020 | Gastroesophageal reflux |
| 606 | HP:0011412 | Ventouse delivery |
|  | HP:0001513 | Obesity |
|  | HP:0000256 | Macrocephaly |
|  | HP:0001999 | Abnormal facial shape |
|  | HP:0008542 | Low-frequency hearing loss |
|  | HP:0001065 | Striae distensae |
|  | HP:0001770 | Toe syndactyly |
|  | HP:0002650 | Scoliosis |
|  | HP:0002020 | Gastroesophageal reflux |
|  | HP:0001263 | Global developmental delay |
|  | HP:0002360 | Sleep disturbance |
|  | HP:0000718 | Aggressive behavior |
|  | HP:0002028 | Chronic diarrhea |
|  | HP:0001985 | Hypoketotic hypoglycemia |
|  | HP:0100512 | Vitamin D deficiency |
|  | HP:0002342 | Intellectual disability, moderate |
|  | HP:0000708 | Behavioural/Psychiatric Abnormality |
|  | HP:0001290 | Generalized hypotonia |
|  | HP:0001251 | Ataxia |
| 608 | HP:0012450 | Chronic constipation |
|  | HP:0001263 | Global developmental delay |
|  | HP:0002020 | Gastroesophageal reflux |
|  | HP:0003128 | Lactic acidosis |
|  | HP:0001943 | Hypoglycemia |
|  | HP:0001251 | Ataxia |
|  | HP:0000733 | Stereotypic behavior |
|  | HP:0000729 | Autism spectrum disorder |
|  | HP:0002307 | Drooling |
|  | HP:0002058 | Myopathic facies |
|  | HP:0011410 | Caesarian section |
| 930 | HP:0011344 | Severe global developmental delay |
|  | HP:0002170 | Intracranial hemorrhage |
|  | HP:0002376 | Developmental regression |
|  | HP:0010818 | Generalized tonic seizures |
|  | HP:0010819 | Atonic seizures |
|  | HP:0011167 | Focal tonic seizures |
|  | HP:0004325 | Decreased body weight |
|  | HP:0004322 | Short stature |
|  | HP:0006349 | Agenesis of permanent teeth |
|  | HP:0000505 | Visual impairment |
|  | HP:0008051 | Abnormality of the retinal pigment epithelium |
|  | HP:0006532 | Recurrent pneumonia |
|  | HP:0002650 | Scoliosis |
|  | HP:0000938 | Osteopenia |
|  | HP:0002239 | Gastrointestinal hemorrhage |
|  | HP:0000107 | Renal cysts |
|  | HP:0000121 | Nephrocalcinosis |
|  | HP:0005208 | Secretory diarrhea |
|  | HP:0001998 | Neonatal hypoglycemia |
|  | HP:0000682 | Abnormality of dental enamel |
|  | HP:0012468 | Chronic acidosis |
|  | HP_0100655 | Thoracic wall defect |
|  | HP:0012469 | Infantile spasms |
|  | HP:0012546 | Skewed maternal X inactivation |
| 2146/ 2156 | HP:0001263 | Global developmental delay |
| 2146 | HP:0001263 | Global developmental delay |
|  | HP:0010864 | Intellectual disability, severe |
|  | HP:0001290 | Generalized hypotonia |
|  | HP:0002072 | Chorea |
|  | HP:0001257 | Spasticity |
|  | HP:0100021 | Cerebral palsy |
|  | HP:0003429 | Hypomyelination |
|  | HP:0001629 | Ventricular septal defect |
|  | HP:0000518 | Cataract |
|  | HP:0000565 | Esotropia |
|  | HP:0000403 | Recurrent otitis media |
|  | HP_0002086 | Abnormality of the respiratory system |
|  | HP:0000767 | Pectus excavatum |
|  | HP:0005750 | Contractures of the joints of the lower limbs |
|  | HP:0002353 | Abnormal EEG |
|  | HP:0000872 | Hashimoto thyroiditis |
|  | HP:0002019 | Constipation |
| 2156 | HP:0001622 | Premature birth |
|  | HP:0001263 | Global developmental delay |
|  | HP:0002342 | Intellectual disability, moderate |
|  | HP:0011398 | Central hypotonia |
|  | HP:0001631 | Defect in the atrial septum |
|  | HP:0001357 | Plagiocephaly |
|  | HP:0000486 | Strabismus |
|  | HP:0000577 | Exotropia |
| 2803 | HP:0010862 | Delayed fine motor development |
|  | HP:0002194 | Delayed gross motor development |
|  | HP:0010864 | Intellectual disability, severe |
|  | HP:0007018 | Attention deficit hyperactivity disorder |
|  | HP:0000708 | Behavioural/Psychiatric Abnormality |
|  | HP:0007319 | Morphological abnormality of the central nervous system |
|  | HP:0002240 | Hepatomegaly |
|  | HP:0012443 | Abnormality of the brain |
| 4306 | HP:0002342 | Intellectual disability, moderate |
|  | HP:0000708 | Behavioural/Psychiatric Abnormality |
|  | HP:0001250 | Seizures |
|  | HP:0001257 | Spasticity |
|  | HP:0007319 | Morphological abnormality of the central nervous system |
| 5356/5357 | HP:0002650 | Scoliosis |
|  | HP:0010862 | Delayed fine motor development |
|  | HP:0002194 | Delayed gross motor development |
|  | HP:0000750 | Delayed speech and language development |
|  | HP:0001328 | Specific learning disability |
|  | HP:0001256 | Intellectual disability, mild |
|  | HP:0001250 | Seizures |
|  | HP:0001332 | Dystonia |
|  | HP:0001257 | Spasticity |
| 5433/ 5434 | HP:0000252 | Microcephaly |
|  | HP:0001263 | Global developmental delay |
| 5433 | HP:0004325 | Decreased body weight |
|  | HP:0000252 | Microcephaly |
|  | HP:0004325 | Decreased body weight |
|  | HP:0004322 | Short stature |
|  | HP:0000252 | Microcephaly |
|  | HP:0000666 | Horizontal nystagmus |
|  | HP:0000378 | Cupped ear |
|  | HP:0009028 | Generalized weakness of limb muscles |
|  | HP:0001263 | Global developmental delay |
|  | HP:0002342 | Intellectual disability, moderate |
|  | HP:0009830 | Peripheral neuropathy |
|  | HP:0003429 | Hypomyelination |
| 5434 | HP:0000252 | Microcephaly |
|  | HP:0100704 | Cortical visual impairment |
|  | HP:0000989 | Pruritus |
|  | HP:0002870 | Obstructive sleep apnea |
|  | HP:0002871 | Central apnea |
|  | HP:0001762 | Talipes equinovarus |
|  | HP:0001738 | Exocrine pancreatic insufficiency |
|  | HP:0002027 | Abdominal pain |
|  | HP:0011471 | Gastrostomy tube feeding in infancy |
|  | HP:0002020 | Gastroesophageal reflux |
|  | HP:0001263 | Global developmental delay |
|  | HP:0100543 | Cognitive impairment |
|  | HP:0002144 | Tethered cord |
|  | HP:0100027 | Recurrent pancreatitis |
|  | HP_0012115 | Hepatitis |
|  | HP:0001319 | Neonatal hypotonia |
| 5628 | HP:0001257 | Spasticity |
|  | HP:0012534 | Dysesthesia |
|  | HP:0002518 | Abnormality of the periventricular white matter |
|  | HP:0002500 | Abnormality of the cerebral white matter |
|  | HP:0100561 | Spinal cord lesions |
|  | HP:0006937 | Impaired distal tactile sensation |
|  | HP:0010829 | Impaired temperature sensation |
|  | HP:0006886 | Impaired distal vibration sensation |
|  | HP:0003487 | Babinski sign |
|  | HP:0006858 | Impaired distal proprioception |
|  | HP:0011448 | Ankle clonus |
|  | HP:0011449 | Knee clonus |
|  | HP:0001288 | Gait disturbance |
|  | HP:0000518 | Cataract |
| 809/ 810 | HP:0002007 | Frontal bossing |
|  | HP:0003510 | Severe short stature |
| 809 | HP:0002514 | Cerebral calcification |
|  | HP:0001263 | Global developmental delay |
|  | HP:0001212 | Prominent fingertip pads |
|  | HP:0009095 | Narrow mouth |
|  | HP:0000319 | Smooth philtrum |
|  | HP:0001622 | Premature birth |
|  | HP:0012470 | Setting-sun eye phenomenon |
|  | HP:0011832 | Pinched nasal tip |
|  | HP:0003510 | Severe short stature |
|  | HP:0001433 | Hepatosplenomegaly |
|  | HP:0001380 | Ligamentous laxity |
|  | HP:0002119 | Ventriculomegaly |
|  | HP:0001511 | Intrauterine growth retardation |
|  | HP:0012510 | Extra-axial cerebrospinal fluid accumulation |
|  | HP:0002007 | Frontal bossing |
|  | HP:0000506 | Telecanthus |
|  | HP:0001252 | Muscular hypotonia |
|  | HP:0004482 | Relative macrocephaly |
|  | HP:0000446 | Narrow nasal bridge |
|  | HP:0008386 | Aplasia/Hypoplasia of the nails |
|  | HP:0012428 | Prominent calcaneus |
|  | HP:0001782 | Bulbous tips of toes |
|  | HP:0000586 | Shallow orbits |
|  | HP:0007598 | Bilateral single transverse palmar creases |
|  | HP:0001043 | Prominent scalp veins |
|  | HP:0000430 | Underdeveloped nasal alae |
|  | HP:0000977 | Soft skin |
|  | HP:0001508 | Failure to thrive |
| 810 | HP:0004325 | Decreased body weight |
|  | HP:0004322 | Short stature |
|  | HP:0001518 | Small for gestational age |
|  | HP:0003510 | Severe short stature |
|  | HP:0005461 | Craniofacial disproportion |
|  | HP:0002007 | Frontal bossing |
|  | HP:0000325 | Triangular face |
|  | HP:0000260 | Wide anterior fontanel |
|  | HP:0000164 | Abnormality of the teeth |
|  | HP:0000670 | Carious teeth |
|  | HP:0000519 | Congenital cataract |
|  | HP:0000577 | Exotropia |
|  | HP:0002750 | Delayed skeletal maturation |
|  | HP:0001382 | Joint hypermobility |
|  | HP:0000047 | Hypospadias |
|  | HP:0002194 | Delayed gross motor development |
|  | HP:0000750 | Delayed speech and language development |
|  | HP:0001290 | Generalized hypotonia |
|  | HP:0002240 | Hepatomegaly |
|  | HP:0008070 | Sparse hair |
| 1647 | HP:0009763 | Limb pain |
|  | HP:0002360 | Sleep disturbance |
|  | HP:0002459 | Dysautonomia |
|  | HP:0002208 | Coarse hair |
|  | HP:0002019 | Constipation |
|  | HP:0012378 | Fatigue |
|  | HP:0012333 | Sudomotor sympathetic dysfunction |
|  | HP:0012343 | Decreased serum ferritin |
| 1757 | HP:0000256 | Macrocephaly |
|  | HP:0000687 | Widely spaced teeth |
|  | HP:0002307 | Drooling |
|  | HP:0002098 | Respiratory distress |
|  | HP:0002835 | Aspiration |
|  | HP:0000767 | Pectus excavatum |
|  | HP:0005750 | Contractures of the joints of the lower limbs |
|  | HP:0001543 | Gastroschisis |
|  | HP:0002015 | Dysphagia |
|  | HP:0002020 | Gastroesophageal reflux |
|  | HP:0001263 | Global developmental delay |
|  | HP:0002342 | Intellectual disability, moderate |
|  | HP:0001332 | Dystonia |
|  | HP:0002072 | Chorea |
|  | HP:0001265 | Hyporeflexia |
|  | HP:0011398 | Central hypotonia |
|  | HP:0002019 | Constipation |
|  | HP:0007687 | Unilateral ptosis |
|  | HP:0012482 | Frontal venous angioma |
|  | HP:0001252 | Muscular hypotonia |
|  | HP:0012487 | Cerebellopontine angle arachnoid cyst |
|  | HP:0002478 | Progressive spastic quadriplegia |
| 2058 | HP:0003484 | Upper limb muscle weakness |
|  | HP:0002273 | Tetraparesis |
|  | HP:0003445 | EMG: neuropathic changes |
|  | HP:0006801 | Hyperactive deep tendon reflexes |
|  | HP:0002072 | Chorea |
|  | HP:0001308 | Tongue fasciculations |
|  | HP:0002366 | Abnormality of the lower motor neuron |
|  | HP:0003722 | Neck flexor weakness |
|  | HP:0002015 | Dysphagia |
|  | HP:0011868 | Sciatica |
|  | HP:0008000 | Decreased corneal reflex |
|  | HP:0007141 | Sensorimotor neuropathy |
|  | HP:0003401 | Paresthesia |
|  | HP:0001260 | Dysarthria |
| 2179 | HP:0001508 | Failure to thrive |
|  | HP:0010535 | Sleep apnea |
|  | HP:0001388 | Joint laxity |
|  | HP:0000938 | Osteopenia |
|  | HP:0005750 | Contractures of the joints of the lower limbs |
|  | HP:0002020 | Gastroesophageal reflux |
|  | HP:0002194 | Delayed gross motor development |
|  | HP:0000750 | Delayed speech and language development |
|  | HP:0000737 | Irritability |
|  | HP:0001315 | Reduced tendon reflexes |
|  | HP:0002033 | Poor suck |
|  | HP:0000349 | Widow's peak |
|  | HP:0000508 | Ptosis |
|  | HP:0001308 | Tongue fasciculations |
|  | HP:0100492 | Joint contractures involving the joints of the feet |
|  | HP:0009698 | Contractures of the proximal interphalangeal joints of the fingers |
|  | HP:0002460 | Distal muscle weakness |
|  | HP:0001004 | Lymphedema |
|  | HP:0001649 | Tachycardia |
|  | HP:0009050 | Quadriceps muscle atrophy |
|  | HP:0000759 | Abnormality of the peripheral nervous system |
|  | HP:0003457 | EMG abnormality |
|  | HP:0003134 | Abnormality of peripheral nerve conduction |
|  | HP:0007739 | Mildly reduced visual acuity |
|  | HP:0009129 | Upper limb amyotrophy |
|  | HP:0003547 | Shoulder girdle muscle weakness |
|  | HP:0006125 | Tapered finger |
|  | HP:0001284 | Areflexia |
|  | HP:0006829 | Severe muscular hypotonia |
|  | HP:0001064 | Hyperhidrosis |
|  | HP:0003382 | Hypertrophic nerve changes |
|  | HP:0000795 | Abnormality of the urethra |
|  | HP:0010044 | Short 4th metacarpal |
|  | HP:0006808 | Cerebral hypomyelination |
|  | HP:0003700 | Generalized amyotrophy |
|  | HP:0012434 | Delayed social development |
|  | HP:0012411 | Premature pubarche |
|  | HP:0007930 | Prominent epicanthal folds |
|  | HP:0012412 | Premature adrenarche |
| 2473 | HP:0001622 | Premature birth |
|  | HP:0002058 | Myopathic facies |
|  | HP:0000565 | Esotropia |
|  | HP:0005946 | Ventilator dependence with inability to wean |
|  | HP:0002650 | Scoliosis |
|  | HP:0002020 | Gastroesophageal reflux |
|  | HP:0000750 | Delayed speech and language development |
|  | HP:0001290 | Generalized hypotonia |
|  | HP:0008959 | Distal upper limb muscle weakness |
|  | HP:0008994 | Proximal muscle weakness in lower limbs |
|  | HP:0002460 | Distal muscle weakness |
|  | HP:0010465 | Precocious puberty in females |
|  | HP:0100512 | Vitamin D deficiency |
|  | HP:0001371 | Flexion contracture |
|  | HP:0002803 | Congenital contractures |
|  | HP:0011471 | Gastrostomy tube feeding in infancy |
| 2542 | HP:0003458 | EMG: myopathic abnormalities |
|  | HP:0001873 | Thrombocytopenia |
|  | HP:0003134 | Abnormality of peripheral nerve conduction |
|  | HP:0003236 | Elevated serum creatine phosphokinase |
|  | HP:0001397 | Hepatic steatosis |
|  | HP:0003701 | Proximal muscle weakness |
|  | HP:0002359 | Frequent falls |
| 2543 | HP:0002194 | Delayed gross motor development |
|  | HP:0001290 | Generalized hypotonia |
|  | HP:0003198 | Myopathy |
|  | HP:0000421 | Epistaxis |
|  | HP:0000967 | Petechiae |
|  | HP:0003391 | Gower sign |
|  | HP:0003540 | Impaired platelet aggregation |
|  | HP:0001873 | Thrombocytopenia |
| 2610 | HP:0000256 | Macrocephaly |
|  | HP:0007302 | Bipolar affective disorder |
|  | HP:0001915 | Aplastic anemia |
|  | HP:0001399 | Hepatic failure |
|  | HP:0006571 | Reduced number of intrahepatic bile ducts |
|  | HP:0002910 | Elevated hepatic transaminases |
|  | HP:0012410 | Pure red cell aplasia |
| 2700 | HP:0002088 | Abnormality of the lung |
|  | HP:0002099 | Asthma |
|  | HP:0006532 | Recurrent pneumonia |
|  | HP:0006515 | Interstitial pneumonitis |
| 2731 | HP:0010864 | Intellectual disability, severe |
|  | HP:0000708 | Behavioural/Psychiatric Abnormality |
|  | HP:0001257 | Spasticity |
|  | HP:0000727 | Frontal lobe dementia |
|  | HP:0010521 | Gait apraxia |
|  | HP:0002500 | Abnormality of the cerebral white matter |
|  | HP:0002352 | Leukoencephalopathy |
|  | HP:0002925 | Thyroid-stimulating hormone excess |
| 2752 | HP:0004323 | Abnormality of body weight |
|  | HP:0001132 | Lens subluxation |
|  | HP:0000978 | Bruising susceptibility |
|  | HP:0001382 | Joint hypermobility |
|  | HP:0002910 | Elevated hepatic transaminases |
|  | HP:0009064 | Generalized lipodystrophy |
| 3138 | HP:0000505 | Visual impairment |
|  | HP:0000479 | Abnormality of the retina |
|  | HP:0001010 | Hypopigmentation of the skin |
|  | HP:0000044 | Hypogonadotrophic hypogonadism |
|  | HP:0001251 | Ataxia |
|  | HP:0002072 | Chorea |
|  | HP:0007319 | Morphological abnormality of the central nervous system |
|  | HP:0001300 | Parkinsonism |
|  | HP:0001107 | Ocular albinism |
| 3404 | HP:0000505 | Visual impairment |
|  | HP:0000639 | Nystagmus |
|  | HP:0008538 | Sensorineural hearing impairment |
|  | HP:0001250 | Seizures |
|  | HP:0001251 | Ataxia |
|  | HP:0001257 | Spasticity |
|  | HP:0000298 | Mask-like facies |
|  | HP:0001300 | Parkinsonism |
|  | HP:0000511 | Vertical supranuclear gaze palsy |
|  | HP:0000649 | Abnormality of vision evoked potentials |
|  | HP:0000657 | Oculomotor apraxia |
|  | HP:0001336 | Myoclonus |
|  | HP:0002344 | Progressive neurologic deterioration |
|  | HP:0002464 | Spastic dysarthria |
|  | HP:0007256 | Abnormality of pyramidal motor function |
|  | HP:0200085 | Limb tremor |
|  | HP:0002172 | Postural instability |
|  | HP:0012049 | Laryngeal dystonia |
|  | HP:0006879 | Pontocerebellar atrophy |
| 3478 | HP:0001557 | Prenatal movement abnormality |
|  | HP:0004322 | Short stature |
|  | HP:0001999 | Abnormal facial shape |
|  | HP:0000678 | Dental crowding |
|  | HP:0000486 | Strabismus |
|  | HP:0000956 | Acanthosis nigricans |
|  | HP:0007502 | Follicular hyperkeratosis |
|  | HP:0007503 | Generalized ichthyosis |
|  | HP:0002808 | Kyphosis |
|  | HP:0003048 | Radial head subluxation |
|  | HP:0000858 | Menstrual irregularities |
|  | HP:0001263 | Global developmental delay |
|  | HP:0002194 | Delayed gross motor development |
|  | HP:0000750 | Delayed speech and language development |
|  | HP:0001265 | Hyporeflexia |
|  | HP:0000589 | Coloboma |
|  | HP:0001328 | Specific learning disability |
|  | HP:0001256 | Intellectual disability, mild |
|  | HP:0002342 | Intellectual disability, moderate |
|  | HP:0010864 | Intellectual disability, severe |
|  | HP:0007018 | Attention deficit hyperactivity disorder |
|  | HP:0000717 | Autism |
|  | HP:0000708 | Behavioural/Psychiatric Abnormality |
|  | HP:0001250 | Seizures |
|  | HP:0001332 | Dystonia |
|  | HP:0001257 | Spasticity |
|  | HP:0100797 | dysplastic toenails |
|  | HP:0012344 | morphea |
|  | HP:0005595 | Generalized hyperkeratosis |
|  | HP:0002032 | Esophageal atresia |
|  | HP:0001543 | Gastroschisis |
|  | HP:0001539 | Omphalocele |
|  | HP:0001290 | Generalized hypotonia |
|  | HP:0007319 | Morphological abnormality of the central nervous system |
|  | HP:0010588 | Premature epimetaphyseal fusion |
|  | HP:0100536 | Abnormality of the fascia |
| 3579 | HP:0001762 | Talipes equinovarus |
|  | HP:0000708 | Behavioural/Psychiatric Abnormality |
|  | HP:0001251 | Ataxia |
|  | HP:0001332 | Dystonia |
|  | HP:0002072 | Chorea |
|  | HP:0001257 | Spasticity |
|  | HP:0007319 | Morphological abnormality of the central nervous system |
| 4245 | HP:0002910 | Elevated hepatic transaminases |
|  | HP:0002024 | Malabsorption |
|  | HP:0002592 | Gastric ulcer |
|  | HP:0100785 | Insomnia |
|  | HP:0002072 | Chorea |
|  | HP:0001891 | Iron deficiency anemia |
|  | HP:0003334 | Elevated circulating catecholamine level |
|  | HP:0000870 | Prolactin excess |
|  | HP:0001962 | Palpitations |
|  | HP:0002013 | Vomiting |
|  | HP:0001662 | Bradycardia |
|  | HP:0001649 | Tachycardia |
|  | HP:0012414 | Duodenal atrophy |
|  | HP:0012452 | Restless legs |
|  | HP:0012343 | Decreased serum ferritin |
|  | HP:0012341 | Microprolactinoma |
|  | HP:0012394 | Iodine contrast allergy |
|  | HP:0000822 | Hypertension |
| 4964 | HP:0001280 | Abnormal gait |
|  | HP:0002135 | Basal ganglia calcification |
|  | HP:0001260 | Dysarthria |
|  | HP:0007120 | Involuntary movements |
| 5376 | HP:0001332 | Dystonia |
|  | HP:0007319 | Morphological abnormality of the central nervous system |
|  | HP:0002135 | Basal ganglia calcification |
|  | HP:0007238 | Nonarteriosclerotic cerebral calcification |
|  | HP:0004963 | Calcification of the aorta |
|  | HP:0002077 | Migraine with aura |
|  | HP:0004934 | Vascular calcification |
|  | HP:0001681 | Angina pectoris |
| 6392 | HP:0000708 | Behavioural/Psychiatric Abnormality |
|  | HP:0001336 | Myoclonus |
|  | HP:0002354 | Memory impairment |
|  | HP:0001250 | Seizures |
|  | HP:0001300 | Parkinsonism |
|  | HP:0100280 | Crohn's disease |

HPID= Human Phenotype number.
