# Supplementary material for: Computational evaluation of exome sequence data using human and model organism phenotypes improves diagnostic efficiency
Source: Genet Med. 2015 Nov 12;18(6):608–17. doi: 10.1038/gim.2015.137 (PMC4916229; doi:10.1038/gim.2015.137)
Supplement: Supplementary Table S2 [file gim2015137x4.doc]

**Table S2. Contribution of human, mouse, and zebrafish phenotypes along with protein-protein association data to the overall performance of Exomiser.**

| Unknown inheritance model (1000 Genomes and ESP freq data) | | | | | | | |
| --- | --- | --- | --- | --- | --- | --- | --- |
|  |  | Human | Mouse | Fish | Human, Mouse | Human, Fish | Human, Mouse, Fish |
| Known associations | Phenotype only | 97.0 | 67.6 | 12.7 | 97.0 | 97.0 | 97.0 |
|  | Phenotype + PPA | 96.9 | 63.8 | 14.7 | 96.9 | 96.9 | 96.9 |
| Novel associations | Phenotype only | 45.4 | 67.6 | 12.7 | 66.3 | 45.9 | 66.5 |
|  | Phenotype + PPA | 67.1 | 63.8 | 14.7 | 73.8 | 67.6 | 73.8 |

Values are shown for the percentage of samples where the causative variant was the top ranked candidate.
