# Supplementary material for: Computational evaluation of exome sequence data using human and model organism phenotypes improves diagnostic efficiency
Source: Genet Med. 2015 Nov 12;18(6):608–17. doi: 10.1038/gim.2015.137 (PMC4916229; doi:10.1038/gim.2015.137)
Supplement: Supplementary Table S3 [file gim2015137x5.doc]

**Table S3. Candidate variants from unsolved cases as ranked by Exomiser**.

| Patient | Gene | Transcript | Predicted Deleteriousness | | | Frequency | | Phenotype | | Match | Scores | | | Rank |
| --- | --- | --- | --- | --- | --- | --- | --- | --- | --- | --- | --- | --- | --- | --- |
|  |  |  | MT | P2 | S | dbS | ESP | Clinical | Matching terms |  | Pheno | Var | ES |  |
| 798 | ZAK | NM_016653.2:  c.[850_854del  AGG];[=] | - | - | - | - | - | Craniosynostosis (HP:0001363) | Craniofacial hyperostosis (HP:0004493) | Proximity to Ectodermal dysplasia 2, Clouston type | 0.44 | 0.90 | 0.39 | 2 |
|  |  |  |  |  |  |  |  | Sparse eyebrow (HP:0000535) | Sparse eyebrow (HP:0000535) |  |  |  |  |  |
|  |  |  |  |  |  |  |  | Conical tooth (HP:0000698) | Abnormality of the teeth (HP:0000164) |  |  |  |  |  |
|  |  |  |  |  |  |  |  | Increased lacrimation (HP:0009926) | Lacrimation abnormality (HP:0000632) |  |  |  |  |  |
|  |  |  |  |  |  |  |  | Abnormality of skin pigmentation (HP:0001000) | Hyperpigmentation of the skin (HP:0000953) |  |  |  |  |  |
| 809/810 | C1orf116 | NM_023938.5:  c.[58G>A];  [861C>G] | 0.70 | 0.06 | 0.22 | - | - | Premature birth (HP:0001622) | Decreased fetal movement (HP:0001558) | Proximity to Minicore myopathy with external ophthalmoplegia | 0.6 | 0.82 | 0.62 | 1 |
|  |  |  |  |  |  |  |  | Global developmental delay (HP:0001263) | Motor delay (HP:0001270) |  |  |  |  |  |
|  |  |  |  |  |  |  |  | Muscular hypotonia (HP:0001252) | Neonatal hypotonia (HP:0001319) |  |  |  |  |  |
|  |  |  |  |  |  |  |  | Ligamentous laxity (HP:0001380) | Ligamentous laxity (HP:0001380) |  |  |  |  |  |
|  |  |  |  |  |  |  |  |  |  |  |  |  |  |  |
| 1166 | WARS2 | NM_015836.3:  c.[37T>G];  [683C>G] | 1.00 | 1.00 | 0.00 | - | - | Tremor (HP:0001337) | Tremors (MP:0000745) | Proximity to mouse mutant involving CARS2 | 0.4 | 0.90 | 0.30 | 8 |
|  |  |  | 1.00 | 0.00 | 1.00 | <0.01 | <0.01 |  |  |  |  |  |  |  |
| 1647 | SELO | NM_031454:c.  [832G>A];  [971A>G] | 1.00 | 0.93 | 0.02 | <0.01 | <0.01 | Constipation (HP:0002019) | Constipation (HP:0002019) | Proximity to Angelman syndrome-like | 0.6 | 0.85 | 0.68 | 3 |
|  |  |  | 1.00 | 0.25 | 0.11 | - | <0.01 | Sleep disturbance (HP:0002360) | Sleep-wake cycle disturbance (HP:0006979) |  |  |  |  |  |
| 1757 | ACER3 | NM_018367.5:c.  [398C>T];  [600-1G>C] | 1.00 | 0.83 | 0.11 | - | - | Muscular hypotonia (HP:0001252) | Muscular hypotonia (HP:0001252) | Proximity to Gaucher disease, perinatal lethal | 0.6 | 0.95 | 0.84 | 1 |
|  |  |  | - | - | - | - | - | Respiratory distress (HP:0002098) | Respiratory distress (HP:0002098) |  |  |  |  |  |
|  |  |  |  |  |  |  |  | Central hypotonia (HP:0011398) | Hypertonia (HP:0001276) |  |  |  |  |  |
|  |  |  |  |  |  |  |  | Dysphagia (HP:0002015) | Dysphagia (HP:0002015) |  |  |  |  |  |
|  |  |  |  |  |  |  |  | Contractures of the joints of the lower limbs (HP:0005750) | Arthrogryposis multiplex congenita (HP:0002804) |  |  |  |  |  |
|  |  |  |  |  |  |  |  | Pectus excavatum (HP:0000767) | Thoracic hypoplasia (HP:0005257) |  |  |  |  |  |
| 2179 | FOXRED2 | NM_001102371.1:  c.[974A>G];  [1008C>G] | 1.00 | 0.98 | 0.00 | <0.01 | <0.01 |  |  | No phenotypic evidence | 0 | 0.84 | 0.29 | 14 |
|  |  |  | 1.00 | 0.05 | 0.23 | - | <0.01 |  |  |  |  |  |  |  |
| 2473 | KLRB1 | NM_002258.2:  c.[397C>T];  [397C>T] | - | - | - | - | - | Esotropia (HP:0000565) | Esotropia (HP:0000565) | Proximity to Gaucher disease, type II | 0.55 | 0.95 | 0.76 | 2 |
|  |  |  |  |  |  |  |  | Ventilator dependence with inability to wean (HP:0005946) | Respiratory insufficiency (HP:0002093)) |  |  |  |  |  |
|  |  |  |  |  |  |  |  | Generalized hypotonia (HP:0001290) | Hypertonia (HP:0001276) |  |  |  |  |  |
|  |  |  |  |  |  |  |  | Flexion contracture (HP:0001371) | Flexion contracture (HP:0001371) |  |  |  |  |  |
|  |  |  |  |  |  |  |  | Gastrostomy tube feeding in infancy (HP:0011471) | Feeding difficulties (HP:0011968) |  |  |  |  |  |
|  |  |  |  |  |  |  |  | Congenital contracture (HP:0002803) | Flexion contracture (HP:0001371) |  |  |  |  |  |
|  |  |  |  |  |  |  |  | Delayed speech and language development (HP:0000750) | Global developmental delay (HP:0001263) |  |  |  |  |  |
|  | CASC3 | NM_007359.4:  c.[1433C>T];  [1433C>T] | 1.00 | 0.49 | 0.12 | <0.01 | <0.01 | Gastroesophageal reflux (HP:0002020) | Gastroesophageal reflux (HP:0002020) | Proximity to Congenital disorder of glycosylation, type Ir | 0.46 | 0.86 | 0.35 | 5 |
|  |  |  |  |  |  |  |  | Esotropia (HP:0000565) | Strabismus (HP:0000486) |  |  |  |  |  |
|  |  |  |  |  |  |  |  | Delayed speech and language development (HP:0000750) | Global developmental delay (HP:0001263) |  |  |  |  |  |
| 2610/3306 | SHPK | NM_013276.2:  c.[990C>G];[=] | 0.98 | 0.03 | 0.25 | - | - | Pure red cell aplasia (HP:0012410) | Pancytopenia (HP:0001876)) | Proximity to Mental retardation, X-linked, syndromic, Martin-Probst type | 0.6 | 0.98 | 0.87 | 1 |
|  |  |  |  |  |  |  |  | Aplastic anemia (HP:0001915) | Pancytopenia (HP:0001876)) |  |  |  |  |  |
| 2700 | FRAS1 | NM_025074.6:  c.[5046C>G];  [7622A>G] | 1.00 | 1.00 | - | <0.01 | <0.01 | Abnormality of the lung(HP:0002088) | Pulmonary hypoplasia (HP:0002089) | Fraser syndrome | 0.58 | 0.79 | 0.51 | 4 |
|  |  |  | 1.00 | 1.00 | 0.05 | <0.01 | <0.01 | Interstitial pneumonitis (HP:0006515) | Pulmonary hypoplasia (HP:0002089) |  |  |  |  |  |
|  |  |  |  |  |  |  |  | Recurrent pneumonia (HP:0006532) | Pulmonary hypoplasia (HP:0002089) |  |  |  |  |  |
|  |  |  |  |  |  |  |  | Asthma (HP:0002099) | Pulmonary hypoplasia (HP:0002089) |  |  |  |  |  |
|  | ALAS2 | NM_000032.4:  c.[1676G>A];[=] | - | 0.00 | 0.35 | - | <0.01 | Abnormality of the lung (HP:0002088) | Respiratory insufficiency (HP:0002093) | Anemia, sideroblastic, X-linked | 0.47 | 0.53 | 0.02 | 8 |
|  |  |  |  |  |  |  |  | Interstitial pneumonitis (HP:0006515) | Respiratory insufficiency(HP:0002093) |  |  |  |  |  |
|  |  |  |  |  |  |  |  | Recurrent pneumonia (HP:0006532) | Respiratory insufficiency  (HP:0002093) |  |  |  |  |  |
|  | STARD8 | NM_001142503.2:  c.[2185C>T];[=] | 1.00 | 1.00 | 0.00 | - | - | Abnormality of the lung (HP:0002088) | abnormal lung morphology (MP:0001175)) | Proximity to mouse mutant of TNS3 | 0.4 | 1.00 | 0.51 | 3 |
|  |  |  |  |  |  |  |  | Interstitial pneumonitis (HP:0006515) | abnormal lung morphology (MP:0001175) |  |  |  |  |  |
|  |  |  |  |  |  |  |  | Recurrent pneumonia (HP:0006532) | abnormal lung morphology (MP:0001175) |  |  |  |  |  |
|  |  |  |  |  |  |  |  | Asthma (HP:0002099) | abnormal lung morphology (MP:0001175) |  |  |  |  |  |
| 2731 | SH3KBP1 | NM_031892.2:  c.[1957A>G];[=] | 1.00 | 0.09 | 0.78 | - | - | Frontal lobe dementia (HP:0000727)) | Frontal lobe dementia (HP:0000727) | Proximity to Leukoencephalopathy, diffuse hereditary, with spheroids | 0.6 | 1.00 | 0.89 | 2 |
|  |  |  |  |  |  |  |  | Spasticity (HP:0001257) | Spasticity (HP:0001257) |  |  |  |  |  |
|  |  |  |  |  |  |  |  | Abnormality of the cerebral white matter (HP:0002500)) | Abnormality of the cerebral white matter (HP:0002500) |  |  |  |  |  |
|  |  |  |  |  |  |  |  | Gait apraxia (HP:0010521) | Apraxia (HP:0002186) |  |  |  |  |  |
|  | DDR1 | NM_013994.2:  c.[526G>A];[=] | 1.00 | 0.99 | - | - | - | Intellectual disability, severe (HP:0010864) | Intellectual disability (HP:0001249)) | Proximity to Oculodentodigital dysplasia | 0.6 | 1.00 | 0.89 | 1 |
|  |  |  |  |  |  |  |  | Spasticity (HP:0001257) | Spasticity (HP:0001257)) |  |  |  |  |  |
|  |  |  |  |  |  |  |  | Abnormality of the cerebral white matter (HP:0002500) | Abnormality of the cerebral white matter (HP:0002500) |  |  |  |  |  |
| 2752 | LTBP1 | NM_206943.2:  c[769G>A];  [2503C>A] | 1.00 | 0.13 | 0.56 | - | - | Joint hypermobility (HP:0001382) | Joint hypermobility (HP:0001382) | Proximity to Pseudochondroplasia | 0.51 | 0.90 | 0.59 | 2 |
|  |  |  | 1.00 | 0.27 | 1.00 | <0.01 | <0.01 |  |  |  |  |  |  |  |
|  | ZYG11A | NM_001004339.2:  c.[67_69delC];[=] | - | - | - | - | - | Elevated hepatic transaminases (HP:0002910) | liver inflammation (MP:0001860) | Proximity to mouse mutant of ZYG11A | 0.45 | 0.95 | 0.52 | 3 |
|  |  |  |  |  |  |  |  | Generalized lipodystrophy (HP:0009064) | decreased total body fat amount (MP:0010025) |  |  |  |  |  |
|  |  |  |  |  |  |  |  | Abnormality of body weight (HP:0004323) | increased susceptibility to weight loss (MP:0010180) |  |  |  |  |  |
| 3138 | IDS | NM_000202.6:  c.[754G>A];[=] | 1.00 | 0.08 | 0.71 | <0.01 | <0.01 | Hypopigmentation of the skin (HP:0001010)) | abnormal retinal pigment epithelium morphology (MP:0005201) | Proximity to mouse mutant of IDS | 0.6 | 0.81 | 0.59 | 6 |
|  |  |  |  |  |  |  |  | Chorea (HP:0002072) | impaired coordination (MP:0001405) |  |  |  |  |  |
|  |  |  |  |  |  |  |  | Ocular albinism(HP:0001107) | abnormal retinal pigment epithelium morphology (MP:0005201) |  |  |  |  |  |
|  |  |  |  |  |  |  |  | Abnormality of the retina (HP:0000479) | abnormal retina morphology (MP:0001325) |  |  |  |  |  |
|  |  |  |  |  |  |  |  | Ataxia (HP:0001251) | impaired coordination (MP:0001405) |  |  |  |  |  |
|  |  |  |  |  |  |  |  | Visual impairment (HP:0000505) | abnormal eye electrophysiology (MP:0005551) |  |  |  |  |  |
| 3404 | ZSCAN10 | NM_032805.1:  c.[1271C>G];  [1271C>G] | 1.00 | 0.86 | 0.00 | 0.01 | <0.01 | Pontocerebellar atrophy (HP:0006879) | Cerebellar atrophy (HP:0001272 | Proximity to Spinocerebellar ataxia 14 | 0.3 | 0.79 | 0.05 | 8 |
|  |  | NM_032805.1:  c.[2080G>T];  [2080G>T] | 1.00 | 0.93 | 0.02 | - | <0.01 | Nystagmus (HP:0000639) | Nystagmus (HP:0000639) |  |  |  |  |  |
|  |  |  |  |  |  |  |  | Laryngeal dystonia (HP:0012049) | Focal dystonia (HP:0004373) |  |  |  |  |  |
|  |  |  |  |  |  |  |  | Progressive neurologic deterioration (HP:0002344) | Mental deterioration (HP:0001268)) |  |  |  |  |  |
|  |  |  |  |  |  |  |  | Abnormality of pyramidal motor function (HP:0007256) | Hyperreflexia (HP:0001347) |  |  |  |  |  |
|  |  |  |  |  |  |  |  | Spastic dysarthria (HP:0002464) | Dysarthria (HP:0001260) |  |  |  |  |  |
| 3579 | DHX38 | NM_014003.3:  c.[987G>A];  [1475C>T] | 1.00 | 0.01 | 0.41 | - | - | Talipes equinovarus (HP:0001762) | Talipes equinovarus (HP:0001762) | Proximity to TARP syndrome | 0.52 | 0.88 | 0.54 | 1 |
|  |  |  | 1.00 | 0.43 | 0.10 | <0.01 | <0.01 |  |  |  |  |  |  |  |
|  | ATXN3L | NM_001135995.1:  c.[76G>T];[=] | - | - | - | <0.01 | <0.01 | Ataxia (HP:0001251 | Decreased vertical activity (MP:0002757) | Mouse mutant involving ATXN3L | 0.51 | 0.79 | 0.32 | 2 |
|  |  |  |  |  |  |  |  | Dystonia (HP:0001332) | Decreased vertical activity (MP:0002757) |  |  |  |  |  |
| 4245 | CCAR1 | NM_018237.2:  c.[2597A>G];[=] | 1.00 | 0.90 | 0.69 | - | - | Elevated hepatic transaminases (HP:0002910) | abnormal liver morphology (MP:0000598)) | Proximity to mouse mutant of IGF2BP1 | 0.4 | 1.00 | 0.51 | 2 |
|  |  |  |  |  |  |  |  | Malabsorption (HP:0002024) | abnormal intestine morphology (MP:0000477) |  |  |  |  |  |
|  |  |  |  |  |  |  |  | Gastric ulcer (HP:0002592) | abnormal intestine morphology (MP:0000477) |  |  |  |  |  |
|  |  |  |  |  |  |  |  | Duodenal atrophy (HP:0012414)) | abnormal intestine morphology (MP:0000477) |  |  |  |  |  |
|  |  |  |  |  |  |  |  | Vomiting (HP:0002013) | abnormal intestine morphology (MP:0000477) |  |  |  |  |  |
|  |  |  |  |  |  |  |  | Iron deficiency anemia (HP:0001891) | anemia (MP:0001577) |  |  |  |  |  |
|  | MYH8 | NM_002472.2:  c.[3686T>C];  [4813G>A] | 1.00 | 0.90 | 0.00 | <0.01 | <0.01 | Elevated circulating catecholamine level (HP:0003334) | Thyroid follicular hyperplasia (HP:0008225) | Carney complex, type 1 | 0.4 | 0.68 | 0.05 | 4 |
|  |  |  | 1.00 | 0.10 | 0.05 | 0.01 | 0.01 | Palpitations (HP:0001962) | Congestive heart failure (HP:0001635) |  |  |  |  |  |
|  |  |  |  |  |  |  |  | Tachycardia (HP:0001649) | Congestive heart failure (HP:0001635) |  |  |  |  |  |
|  |  |  |  |  |  |  |  | Hypertension (HP:0000822) | Congestive heart failure (HP:0001635) |  |  |  |  |  |
|  |  |  |  |  |  |  |  | Bradycardia (HP:0001662) | Congestive heart failure (HP:0001635) |  |  |  |  |  |
|  |  |  |  |  |  |  |  | Prolactin excess (HP:0000870) | Growth hormone excess (HP:0000845) |  |  |  |  |  |

Abbreviations: MT = MutationTaster, P2 = Polyphen2, S = SIFT, dbS = dbSNP, ESP = Exome Sequencing Project, Pheno = Phenotype scoreVar = Variant score, ES = Exomiser Score, Rank = The rank of the variant within the perspective filtered family VCF file, **Bolded text** indicates variants that are referenced in the text, but are not considered a strong variant.
