# Supplementary material for: Computational evaluation of exome sequence data using human and model organism phenotypes improves diagnostic efficiency
Source: Genet Med. 2015 Nov 12;18(6):608–17. doi: 10.1038/gim.2015.137 (PMC4916229; doi:10.1038/gim.2015.137)
Supplement: Supplementary Table S4 [file gim2015137x6.doc]

**Table S4.** Variant ranking for siblings in UDP_606 when prioritized by Exomiser under different assumptions of affected status.

| Patient | Variant | Gene | 606*/608* | 606*/608 | 608*/606 | 606* | 608* |
| --- | --- | --- | --- | --- | --- | --- | --- |
| 606/608 | NM_002591.3:  c.[134T>C];  [134T>C] | PCK1 | 1 | - | - | 3 | 2 |
| 606 | NM_030665.3  :c.[2273G>A];[=] | RAI1 | - | 1 | - | 1 | - |
| 608 | NM_000834.3:  c.[1238A>G];[=] | GRIN2B | - | - | 3 | - | 5 |

“*”= patient was assumed to be affected, “-”= variant was filtered away by Mendelian filter. Column headers indicate the affected status of the UDP_606 family siblings in each analysis. Mean gene count before ﬁltering was 8388 +/- 882 (s.d.m.); mean post-filtering gene count was 895 +/- 152 (unknown inheritance); 624 +/- 151 (AD inheritance); 368 +/- 25 (AR inheritance).
