# Supplementary material for: Computational evaluation of exome sequence data using human and model organism phenotypes improves diagnostic efficiency
Source: Genet Med. 2015 Nov 12;18(6):608–17. doi: 10.1038/gim.2015.137 (PMC4916229; doi:10.1038/gim.2015.137)
Supplement: Supplementary Table S5 [file gim2015137x7.docx]

**Table 2. Exomiser variant ranking for 9 families with diagnoses.**

| Patient | Gene | Transcript | Predicted Deleteriousness | | | Frequency | | Phenotype | | Match | Scores | | | Rank out of x filtered genes |
| --- | --- | --- | --- | --- | --- | --- | --- | --- | --- | --- | --- | --- | --- | --- |
|  |  |  | MT | P2 | S | dbS | ESP | Clinical | Matching terms |  | Pheno | Var | ES |  |
| 579/577 | PIGT | NM_015937.4:c.[1342C>T];[918_918insT] | 1.00 | 1.00 | 0.00 | - | - | Prominent epicanthal folds (HP:0007930)) | Long palpebral fissure (HP:0000637) | Proximity to PIGO- Hyperphosphatasia with mental retardation syndrome 2 | 0.60 | 0.98 | 0.87 | 1^st^ out of 34 genes |
|  |  |  | 1.00 | 1.00 | 0.00 | - | - | Status epilepticus (HP:0002133) | Seizure  (HP:0001250) |  |  |  |  |  |
|  |  |  |  |  |  |  |  | Epicanthus inversus (HP:0000537) | Long palpebral fissure (HP:0000637) |  |  |  |  |  |
|  |  |  |  |  |  |  |  | Severe global developmental delay (HP:0011344) | Delayed speech and language development (HP:0000750) |  |  |  |  |  |
|  |  |  |  |  |  |  |  | Global brain atrophy (HP:0002283) | Microcephaly (HP:0000252) |  |  |  |  |  |
|  |  |  |  |  |  |  |  | Tented upper lip vermilion (HP:0010804) | Tented upper lip vermilion (HP:0010804) |  |  |  |  |  |
|  |  |  |  |  |  |  |  | Ventricular septal defect (HP:0001629) | Defect in the atrial septum (HP:0001631) |  |  |  |  |  |
| 606/608 | PCK1 | NM_002591.3:c.[134T>C];[134T>C] | 1.00 | 0.94 | 0.01 | - | - | Hypoketotic Hypoglycemia (HP:0001985) | Hypoketotic hypoglycemia (HP:0001985) | Proximity to HADHB - Trifunctional protein deficiency | 0.60 | 1.00 | 0.89 | 1^st^ out of 19 genes |
|  |  |  |  |  |  |  |  | Global developmental delay (HP:0001263) | Global developmental delay (HP:0001263) |  |  |  |  |  |
|  |  |  |  |  |  |  |  | Generalized hypotonia (HP:0001290) | Muscular hypotonia (HP:0001252) |  |  |  |  |  |
| 606 | RAI1 | NM_030665.3:c.[2273G>A];[=] | - | - | - | - | - | Macrocephaly (HP:0000256) | Microcephaly (HP:0000252) | Smith-Magenis  syndrome | 0.70 | 0.95 | 0.93 | 1^st^ out of 62 genes |
|  |  |  |  |  |  |  |  | Abnormal facial shape (HP:0001999) | Malar flattening (HP:0000272) |  |  |  |  |  |
|  |  |  |  |  |  |  |  | Chronic diarrhea  (HP:0002028) | Constipation (HP:0002019) |  |  |  |  |  |
|  |  |  |  |  |  |  |  | Global developmental delay (HP:0001263) | Global developmental delay (HP:0001263) |  |  |  |  |  |
|  |  |  |  |  |  |  |  | Generalized hypotonia (HP:0001290) | Generalized hypotonia (HP:0001290) |  |  |  |  |  |
|  |  |  |  |  |  |  |  | Obesity (HP:0001513) | Obesity (HP:0001513) |  |  |  |  |  |
|  |  |  |  |  |  |  |  | Low-frequency hearing loss (HP:0008542) | Conductive hearing impairment (HP:0000405) |  |  |  |  |  |
|  |  |  |  |  |  |  |  | Aggressive behavior (HP:0000718) | Self-mutilation (HP:0000742) |  |  |  |  |  |
|  |  |  |  |  |  |  |  | Sleep disturbance (HP:0002360) | Sleep Disturbance (HP:0002360) |  |  |  |  |  |
|  |  |  |  |  |  |  |  | Scoliosis (HP:0002650) | Scoliosis (HP:0002650) |  |  |  |  |  |
|  |  |  |  |  |  |  |  | Toe syndactyly (HP:0001770) | Toe syndactyly (HP:0001770) |  |  |  |  |  |
|  |  |  |  |  |  |  |  | Behavioural/Psychiatric abnormality (HP:0000708) | Stereotypic behavior (HP:0000733) |  |  |  |  |  |
| 608 | GRIN2B | NM_000834.3:c.[1238A>G];[=] | 1.00 | 1.00 | 0.00 | - | - | Ataxia (HP:0001251) | Hypoactivity (MP:0001402)) | Proximity to mouse mutant of GRK5 | 0.57 | 1.00 | 0.86 | 1^st^ out of 54 genes |
|  |  |  |  |  |  |  |  | Gastroesophageal reflux (HP:0002020) | Abnormal muscle contractility (MP:0005620) |  |  |  |  |  |
|  |  |  |  |  |  |  |  | Myopathic facies (HP:0002058) | Abnormal muscle contractility (MP:0005620) |  |  |  |  |  |
|  |  |  |  |  |  |  |  | Drooling (HP:0002307) | Increased salivation (MP:0000622) |  |  |  |  |  |
| 930/929 | SMS | NM_004595.4:c.[443A>G];[0] | 1.00 | 0.99 | 0.00 | - | - | Osteopenia (HP:0000938) | Decreased bone mineral density (MP:0000063) | Mouse mutant involving SMS | 0.45 | 1.00 | 0.89 | 1^st^ out of 25 genes |
|  |  |  |  |  |  |  |  | Short stature (HP:0004322) | decreased body length (MP:0001258) |  |  |  |  |  |
|  |  |  |  |  |  |  |  | Neonatal hypoglycemia (HP:0001998) | - decreased circulating glucose level (MP:0005560) |  |  |  |  |  |
|  |  |  |  |  |  |  |  | Chronic acidosis (HP:0012468) | decreased circulating potassium level (MP:0005628) |  |  |  |  |  |
|  |  |  |  |  |  |  |  | Decreased body weight (HP:0004325) | Decreased body weight (HP:0004325) |  |  |  |  |  |
| 2146/2156 | MED23 | NM_004830.3:c.[3638A>G];[3988C>T] | - | - | - | - | - | Esotropia (HP:0000565) | Strabismus (HP:0000486) | Proximity to NIPBL - Brachmann-de Lange syndrome | 0.6 | 0.98 | 0.87 | 2^nd^ out of 12 genes |
|  |  |  | - | - | - | - | - | Generalized hypotonia (HP:0001290) | Muscular hypotonia (HP:0001252)) |  |  |  |  |  |
|  |  |  |  |  |  |  |  | Cerebral palsy (HP:0100021)) | Hypertonia (HP:0001276) |  |  |  |  |  |
|  |  |  |  |  |  |  |  | Recurrent otitis media (HP:0000403) | Conductive hearing impairment (HP:0000405) |  |  |  |  |  |
|  |  |  |  |  |  |  |  | Spasticity (HP:0001257) | Hypertonia (HP:0001276) |  |  |  |  |  |
|  |  |  |  |  |  |  |  | Contractures of the joints of the lower limbs (HP:0005750) | Limitation of joint mobility (HP:0001376) |  |  |  |  |  |
|  |  |  |  |  |  |  |  | Ventricular septal defect (HP:0001629) | Ventricular septal defect (HP:0001629) |  |  |  |  |  |
|  |  |  |  |  |  |  |  | Cataract (HP:0000518) | Cataract (HP:0000518) |  |  |  |  |  |
|  |  |  |  |  |  |  |  | Pectus excavatum (HP:0000767) | Pectus excavatum (HP:0000767) |  |  |  |  |  |
| 2803/2805 | NAGLU | NM_000263.3:  c.[1949G>A];  [1946G>T] | 1.00 | 0.99 | 0.00 | - | - | Attention deficit hyperactivity disorder (HP:0007018) | Hyperactivity (HP:0000752) | Mucopolysaccharidosis type IIIB (Sanfilippo B) | 0.67 | 1.00 | 0.94 | 1^st^ out of 6 genes |
|  |  |  | 1.00 | 1.00 | 0.02 | - | - | Hepatomegaly (HP:0002240) | Hepatomegaly (HP:0002240) |  |  |  |  |  |
|  |  |  |  |  |  |  |  | Behavioural/Psychiatric abnormality (HP:0000708) | Aggressive behavior (HP:0000718) |  |  |  |  |  |
| 4306 | AARS2 | NM_020745.3:c.[595C>T];[2681C>A] | - | - | - | - | - | Spasticity (HP:0001257) | Spastic tetraparesis (HP:0001285) | Proximity to EARS2 Combined oxidative phosphorylation deficiency 12 | 0.37 | 0.91 | 0.25 | 7^th^ out of 60 genes |
|  |  |  | 1.00 | 0.96 | 0.00 | - | <0.01 | Seizures (HP:0001250) | Seizures (HP:0001250) |  |  |  |  |  |
| 5356/5357 | RNASEH2B | NM_024570.3:c.[529G>A];[529G>A] | 0.93 | 0.89 | 0.61 | - | <0.01 | Seizures (HP:0001250) | Seizures (HP:0001250) | Aicardi- Goutières syndrome | 0.60 | 0.76 | 0.49 | 2^nd^ out of 6 genes |
|  |  |  |  |  |  |  |  | Spasticity (HP:0001257) | Spasticity (HP:0001257) |  |  |  |  |  |
|  |  |  |  |  |  |  |  | Delayed gross motor development (HP:0002194) | Global developmental delay (HP:0001263) |  |  |  |  |  |
|  |  |  |  |  |  |  |  | Intellectual disability, mild (HP:0001256) | Intellectual disability, profound (HP:0002187) |  |  |  |  |  |
|  |  |  |  |  |  |  |  | Dystonia (HP:0001332) | Dystonia (HP:0001332) |  |  |  |  |  |
|  |  |  |  |  |  |  |  | Delayed speech and language development (HP:0000750) | Global developmental delay (HP:0001263) |  |  |  |  |  |
|  |  |  |  |  |  |  |  | Delayed fine motor development (HP:0010862) | Global developmental delay (HP:0001263) |  |  |  |  |  |
| 5433/  5434 | ERCC6 | NM_000124.3:c.[543+4delA];[2008C>T] | 1.00 | 1.00 | 0.00 | - | - | Horizontal nystagmus (HP:0000666) | Nystagmus (HP:0000639) | Cockayne syndrome, type B | 0.71 | 0.50 | 0.21 | 4^th^ out of 26 genes |
|  |  |  | - | - | - | - | - | Microcephaly (HP:0000252) | Microcephaly (HP:0000252) |  |  |  |  |  |
|  |  |  |  |  |  |  |  | Short stature (HP:0004322) | Severe short stature (HP:0003510) |  |  |  |  |  |
|  |  |  |  |  |  |  |  | Peripheral neuropathy (HP:0009830) | Polyneuropathy (HP:0001271) |  |  |  |  |  |
|  |  |  |  |  |  |  |  | Intellectual disability, moderate (HP:0002342) | Intellectual disability (HP:0001249) |  |  |  |  |  |
|  |  |  |  |  |  |  |  | Generalized weakness of limb muscles (HP:0009028) | Muscle weakness (HP:0001324) |  |  |  |  |  |
|  |  |  |  |  |  |  |  | Cupped ear (HP:0000378) | Abnormality of the pinna (HP:0000377) |  |  |  |  |  |
|  |  |  |  |  |  |  |  | CNS hypomyelination (HP:0003429) | Patchy demyelination of subcortical white matter (HP:0002545) |  |  |  |  |  |
|  |  |  |  |  |  |  |  | Decrease body weight (HP:0004325) | Severe failure to thrive (HP:0001525) |  |  |  |  |  |
| 5628 | DARS | NM_001349.2:c.[839A>T];[1099G>C] | 1.00 | 0.99 | 0.00 | - | <0.01 | Abnormality of the cerebral white matter (HP:0002500) | Hypoplasia of the corpus callosum (HP:0002079) | Hypomyelination with brainstem and spinal cord involvement and leg spasticity | 0.54 | 0.93 | 0.71 | 2^nd^ out of 71 genes |
|  |  |  | 1.00 | 1.00 | 0.00 | - | - | Babinski sign (HP:0003487) | Babinski sign (HP:0003487) |  |  |  |  |  |
|  |  |  |  |  |  |  |  | Abnormality of the periventricular white matter (HP:0002518) | Leukoencephalopathy (HP:0002352) |  |  |  |  |  |
|  |  |  |  |  |  |  |  | Spasticity (HP:0001257) | Spasticity (HP:0001257) |  |  |  |  |  |

MT = MutationTaster, P2 = Polyphen2, S = SIFT, dbS = frequency in dbSNP, ESP = frequency in Exome Sequencing Project, Pheno = Phenotype score, Var = Variant score, ES = Exomiser Score, Rank = The rank of the variant within the perspective filtered family VCF file that contains combined output from compound-heterozygous, homozygous, heterozygous and X-linked filtering.
