# Supplementary material for: Computational evaluation of exome sequence data using human and model organism phenotypes improves diagnostic efficiency
Source: Genet Med. 2015 Nov 12;18(6):608–17. doi: 10.1038/gim.2015.137 (PMC4916229; doi:10.1038/gim.2015.137)
Supplement: Supplementary Methods [file gim2015137x8.doc]

**Computational evaluation of exome sequence data using human and model organism phenotypes improves diagnostic efficiency**

**Evaluation of exome data using human and model organism phenotypes**

William P. Bone^1^, Nicole L. Washington, Ph.D.^2^, Orion J. Buske^3,4^, David R. Adams, M.D.,Ph.D.^1,5^, Joie Davis^1^, David Draper^1^, Elise D. Flynn^1^, Marta Girdea^3,4^, Rena Godfrey^1^, Gretchen Golas^1^, Catherine Groden^1^, Julius Jacobsen^6^, Sebastian Köhler, Ph.D.^7^, Elizabeth M. J. Lee^1^, Amanda E. Links^1^, Thomas C. Markello, M.D.,Ph.D.^1^, Christopher J. Mungall, Ph.D.^2^, Michele Nehrebecky^1^, Peter N. Robinson, M.D.,Ph.D.^7^, Murat Sincan, M.D.Ph.D.^1^, Ariane G. Soldatos, M.D.^1^, Cynthia J. Tifft, M.D.,Ph.D.^1,5^, Camilo Toro, M.D.^1^, Heather Trang^3,4^, Elise Valkanas^1^, Nicole Vasilevsky, Ph.D.^8^, Colleen Wahl^1^, Lynne A. Wolfe^1^, Cornelius F. Boerkoel, M.D.,Ph.D.^1^, Michael Brudno, Ph.D.^3,4^, Melissa A. Haendel, Ph.D.^8^, William A. Gahl, M.D.,Ph.D.^1,5^, Damian Smedley, Ph.D.^6^

^1^Undiagnosed Diseases Program, Common Fund, Office of the Director, National Institutes of Health, Bethesda, Maryland, United States of America

^2^Genomics Division, Lawrence Berkeley National Laboratory, Berkeley, California, United States of America

^3^Centre for Computational Medicine Hospital for Sick Children, Toronto, Ontario, Canada

^4^Department of Computer Science, University of Toronto, Toronto Ontario, Canada

^5^Medical Genetics Branch, National Human Genome Research Institute, Bethesda, Maryland, United States of America

^6^Mouse Informatics group, Wellcome Trust Sanger Institute, Hinxton, United Kingdom

^7^Institute for Medical Genetics and Human Genetics, Charité-Universitätsmedizin Berlin, Berlin, Germany

^8^Library; and Department of Medical Informatics and Epidemiology, Oregon Health & Science University, Portland, Oregon, United States of America

**Corresponding Author:**

Damian Smedley

Wellcome Trust Sanger Institute,

Hinxton, Cambridge, CB10 1SA, UK,

Telephone: +44 (0)1223 834244

Fax: +44 (0)1223 494919

[ds5@sanger.ac.uk](mailto:ds5@sanger.ac.uk).

**MATERIALS AND METHODS**

**Exome sequencing**

Genomic DNA was extracted from whole blood using the Gentra Puregene Blood kit (Qiagen, Valencia, CA). Patient exome data were aligned using one of two methods. Sequence reads were aligned to a human reference sequence (UCSC assembly hg19/NCBI build 37) using Novoalign, and genotypes were called using the Most Probable Genotype algorithm [^1^](#_ENREF_1). Alternatively, sequence reads were aligned and genotyped using the UDP’s DiploidAlign pipeline. Briefly, BEAGLE software version 3 (RRID:nlx_154238) was used to generate a phased and imputed Variant Call Format (VCF) file from SNP chip data of the parents and offspring and 1000 Genomes HapMap data [^2^](#_ENREF_2). The VCF file was then used by vcf2diploid version 0.2.3 to modify the human reference and create a maternal reference and a paternal reference, which are concatenated together to generate a parental reference [^3^](#_ENREF_3). Patient short reads were aligned with Novoalign version 2.08.03 (<http://www./novocraft.com/main/downloadpage.php>) to each of the three reference sequences and were lifted back over to the standard human reference using custom Java code. Bam files were recalibrated and genotyped by HaplotypeCaller according to GATK Best Practices using GATK v2.5-2 (<http://www.broadinstitute.org/gatk/>) [^4^](#_ENREF_4). Variants in the VCF ﬁles were annotated relative to RefSeq transcripts using ANNOVAR [^5^](#_ENREF_5).

**Filtration of Exome Variants**

Variants listed in the VCF files were filtered for rarity, segregation, deleteriousness, and quality. We defined rare as an allele frequency of <6% in the subpopulations of the UDP cohort used in this experiment (one cohort being the NISC aligned data and the other being the Diploid Aligned cohort) and as <2% in the Exome Sequencing Project v.0.0.20 and dbSNP build 137 (RRID:nif-0000-02734) databases [^6^](#_ENREF_6). From these rare variants, we selected those segregating with disease according to autosomal recessive, *de novo* dominant, and X-linked recessive inheritance models. We then excluded biallelic variants that (excluding the affected individuals of the family) occurred in homozygosity more than once in the UDP cohort and *de novo* variants that (excluding the affected individuals of the family) occurred more than once in the UDP cohort. From the remaining variants, we selected those annotated as nonsynonymous, frame shift, premature stop, loss of start codon, loss of stop codon, or splicing mutations. This list of variants was then submitted to Exomiser for ranking.

**Exomiser data sources**

Exomiser uses an underlying database that stores 1) known disease-gene associations 2) disease-phenotype associations 3) mouse and zebrafish gene-phenotype associations 4) intra- and inter-species phenotype matches computed via OwlSim v1, 5) orthology mappings, 6) predicted pathogenicity for all non-synonymous coding variants and 7) allele frequency data for known human variants. All data and ontology files were downloaded 1^st^ August 2014. Variant population frequency data were downloaded from the Phase I 1000 Genomes Project component of dbSNP [^7^](#_ENREF_7) and from the Exome Variant Server (6500 version; NHLBI GO Exome Sequencing Project 2013). Predicted pathogenicities from SIFT [^8^](#_ENREF_8), Polyphen2 [^9^](#_ENREF_9), and MutationTaster [^10^](#_ENREF_10) were extracted from dbNSFP v2.4 [^11^](#_ENREF_11). Associations between genes and Mendelian diseases were extracted from the Online Mendelian Inheritance in Man (OMIM) morbidmap [^12^](#_ENREF_12) and Orphanet [^13^](#_ENREF_13). Phenotypic annotations to human diseases from OMIM and Orphanet are available from the Human Phenotype Ontology (HPO) resource (<http://www.human-phenotype-ontology.org/>).Orthology mappings, Mammalian Phenotype Ontology (MPO) annotations for mouse models [^14^](#_ENREF_14) were downloaded from the Mouse Genome Informatics (MGI) ftp site [^15^](#_ENREF_15) and the Sanger Mouse Portal (<http://www.sanger.ac.uk/mouseportal>). Zebrafish annotations were obtained from the Zebrafish Model Organism database (ZFIN) in entity-quality format using the Zebrafish Anatomical Ontology [^16^](#_ENREF_16) Gene Ontology [^17^](#_ENREF_17), and PATO ontology of qualities; these were converted to a combined Zebrafish Phenotype (ZP) term (see the Monarch Initiative website). Only gene-phenotype associations involving a single gene disruption in a wild-type environment were used for the mouse and fish models.

**Evaluation of Exome Variants following Exomiser Ranking**

Using the Integrative Genome Viewer (<https://www.broadinstitute.org/igv/home>) [^19^](#_ENREF_19), we reviewed the quality of alignment and genotype of the highest ranked variants. To ensure reliable genotype calls, variants were required to have a read depth >10 and needed to have less than a 3:1 allelelic skewing. Triallelic variants and variants called in a locus containing five or more SNPs per 50 base pairs were excluded as alignment artifacts. To be considered a viable disease-associated candidate, the frequency of other apparently deleterious variants within the gene and of the appropriate zygosity had to be <2% of the entire UDP cohort.

**1.** Teer JK, Bonnycastle LL, Chines PS, et al. Systematic comparison of three genomic enrichment methods for massively parallel DNA sequencing. *Genome Res.* Oct 2010;20(10):1420-1431.

**2.** Browning BL, Browning SR. A unified approach to genotype imputation and haplotype-phase inference for large data sets of trios and unrelated individuals. *Am J Hum Genet.* Feb 2009;84(2):210-223.

**3.** Rozowsky J, Abyzov A, Wang J, et al. AlleleSeq: analysis of allele-specific expression and binding in a network framework. *Mol Syst Biol.* 2011;7:522.

**4.** McKenna A, Hanna M, Banks E, et al. The Genome Analysis Toolkit: a MapReduce framework for analyzing next-generation DNA sequencing data. *Genome Res.* Sep 2010;20(9):1297-1303.

**5.** Wang K, Li M, Hakonarson H. ANNOVAR: functional annotation of genetic variants from high-throughput sequencing data. *Nucleic Acids Res.* Sep 2010;38(16):e164.

**6.** Biesecker LG, Mullikin JC, Facio FM, et al. The ClinSeq Project: piloting large-scale genome sequencing for research in genomic medicine. *Genome Res.* Sep 2009;19(9):1665-1674.

**7.** Sherry ST, Ward MH, Kholodov M, et al. dbSNP: the NCBI database of genetic variation. *Nucleic Acids Res.* Jan 1 2001;29(1):308-311.

**8.** Ng PC, Henikoff S. Accounting for human polymorphisms predicted to affect protein function. *Genome Res.* Mar 2002;12(3):436-446.

**9.** Adzhubei IA, Schmidt S, Peshkin L, et al. A method and server for predicting damaging missense mutations. *Nat Methods.* Apr 2010;7(4):248-249.

**10.** Schwarz JM, Rodelsperger C, Schuelke M, Seelow D. MutationTaster evaluates disease-causing potential of sequence alterations. *Nat Methods.* Aug 2010;7(8):575-576.

**11.** Liu X, Jian X, Boerwinkle E. dbNSFP: a lightweight database of human nonsynonymous SNPs and their functional predictions. *Hum Mutat.* Aug 2011;32(8):894-899.

**12.** Amberger J, Bocchini C, Hamosh A. A new face and new challenges for Online Mendelian Inheritance in Man (OMIM(R)). *Hum Mutat.* May 2011;32(5):564-567.

**13.** Maiella S, Rath A, Angin C, Mousson F, Kremp O. Orphanet et son réseau : où trouver une information validée sur les maladies rares. *Revue Neurologique.* 2013;169, Supplement 1(0):S3-S8.

**14.** Smith CL, Goldsmith CA, Eppig JT. The Mammalian Phenotype Ontology as a tool for annotating, analyzing and comparing phenotypic information. *Genome Biol.* 2005;6(1):R7.

**15.** Bult CJ, Eppig JT, Blake JA, Kadin JA, Richardson JE. The mouse genome database: genotypes, phenotypes, and models of human disease. *Nucleic Acids Res.* Jan 2013;41(Database issue):D885-891.

**16.** Van Slyke CE, Bradford YM, Westerfield M, Haendel MA. The zebrafish anatomy and stage ontologies: representing the anatomy and development of Danio rerio. *J Biomed Semantics.* 2014;5(1):12.

**17.** Mungall CJ, Gkoutos GV, Smith CL, Haendel MA, Lewis SE, Ashburner M. Integrating phenotype ontologies across multiple species. *Genome Biol.* 2010;11(1):R2.

**18.** Gkoutos GV, Green EC, Mallon AM, Hancock JM, Davidson D. Using ontologies to describe mouse phenotypes. *Genome Biol.* 2005;6(1):R8.

**19.** Thorvaldsdottir H, Robinson JT, Mesirov JP. Integrative Genomics Viewer (IGV): high-performance genomics data visualization and exploration. *Brief Bioinform.* Mar 2013;14(2):178-192.
